# RNA-binding protein complex LIN28/MSI2 enhances cancer stem cell-like properties by modulating Hippo-YAP1 signaling and independently of Let-7

**DOI:** 10.1038/s41388-022-02198-w

**Published:** 2022-01-31

**Authors:** Hailin Zou, Juan Luo, Yibo Guo, Yuhong Liu, Yun Wang, Liang Deng, Peng Li

**Affiliations:** 1 Scientific Research Center, The Seventh Affiliated Hospital of Sun Yat-sen University, Shenzhen, 518107 Guangdong People’s Republic of China; 2 Department of General Surgery, The Seventh Affiliated Hospital of Sun Yat-sen University, Shenzhen, 518107 Guangdong People’s Republic of China; 3 Guangdong Provincial Key Laboratory of Digestive Cancer Research, The Seventh Affiliated Hospital of Sun Yat-sen University, No. 628 Zhenyuan Road, Shenzhen, 518107 Guangdong People’s Republic of China

**Keywords:** Breast cancer, Mechanisms of disease

## Abstract

The RNA binding protein LIN28 directly modulates the stability and translation of target mRNAs independently of Let-7; however, the key downstream targets of LIN28 in this process are largely unknown. Here, we revealed that Hippo signaling effector YAP1 functioned as a key downstream regulator of LIN28 to modulate the cancer stem cell (CSC)-like properties and tumor progressions in triple negative breast cancer (TNBC). LIN28 was overexpressed in BC tissues and cell lines, and significantly correlated with poorer overall survivals in patients. Ectopic LIN28 expression enhanced, while knockdown of LIN28A inhibited the CSC-like properties, cell growth and invasive phenotypes of TNBC cells in vitro and in vivo. Transcriptome analysis demonstrated LIN28 overexpression significantly induced the expressions of YAP1 downstream genes, while reduced the transcripts of YAP1 upstream kinases, such as MST1/2 and LATS1/2, and knockdown of LIN28A exhibited the opposite effects. Furthermore, constitutive activation of YAP1 in LIN28 knockdown TNBC cells could rescue the cell growth and invasive phenotypes in vitro and in vivo. Mechanistically, instead of the dependence of Let-7, LIN28 recruited RNA binding protein MSI2 in a manner dependent on the LIN28 CSD domain and MSI2 RRM domain, to directly induce the mRNA decay of YAP1 upstream kinases, leading to the inhibition of Hippo pathway and activation of YAP1, which eventually gave rise to increased CSC populations, enhanced tumor cell growth and invasive phenotypes. Accordingly, co-upregulations of LIN28 and MSI2 in TNBC tissues were strongly associated with YAP1 protein level and tumor malignance. Taken together, our findings unravel a novel LIN28/MSI2-YAP1 regulatory axis to induce the CSC-like properties, tumor growth and metastasis, independently of Let-7, which may serve as a potential therapeutic strategy for the treatment of a subset of TNBC with LIN28 overexpression.

## Introduction

CSCs are a small population of cells with properties of self-renewal and multipotency, which are believed to contribute to the development and overall aggressiveness of the recurrent or metastatic lesions [1, 2]. TNBC is the most aggressive BC subtypes with no effective standard therapy [3, 4]. Breast CSCs are mainly enriched in primary TNBCs and highly related to the ‘triple-negative’ state and unfavorable prognosis in BC patients [5, 6]. Therefore, targeting CSC regulation might be a promising strategy for curing the TNBC.

The RNA-binding protein LIN28 is initially identified as a developmental timing regulator in *C.elegans*, and it has two homologs LIN28A/B in mammals [7, 8]. Accumulating studies have revealed that LIN28 is a master regulator for controlling the pluripotency of stem cells, and the stemness of cancer cells [9–12]. For example, LIN28 combined with a cocktail of core reprogramming factors, like OCT4 and SOX2 was able to promote both mouse and human iPSC reprogramming efficiency by upregulating numerous cell-cycle and cell growth regulators [13, 14]. In addition, human LIN28A/B were upregulated in a spectrum of tumors (~15%), and the elevated expression of them was often associated with increased cancer aggression and poor prognoses [15]. Transgenic overexpression of LIN28 in mice has been shown to induce various kinds of tumors, including T cell lymphoma, neuroblastoma, and intestinal adenocarcinoma [16–18]. Emerging evidences also showed that LIN28 was highly enriched in breast CSC populations and played an essential role in maintaining CSC properties [12, 19], thereby expanding its function as a regulator of CSC stemness. In all these processes mentioned above, Let-7 miRNA family has identified to be the downstream target of LIN28, by which to inhibit Let-7 maturation [20, 21]. Indeed, blockage of let-7 biogenesis and subsequent de-repression of Let-7 target genes by LIN28 have repeatedly proven to be the underlying mechanisms for LIN28-induced cancer progression and metastasis [22]. Besides, emerging evidences indicated that LIN28 could function as a transcriptional or translational regulator independently of the Let-7 [10, 23–26]. However, the key downstream targets of LIN28 in this process are not clear.

In present study, we identified the Hippo pathway effector YAP1 as the key downstream target of LIN28 to induce the CSC-like properties, tumor growth and metastasis in TNBC, which is independent of Let-7. And we further demonstrated that MSI2 could be recruited directly by LIN28 to mediate the mRNA degradations of YAP1 upstream kinases, thereby leading to the Hippo pathway inhibition and YAP1 activation. These findings revealed a novel molecular mechanism that the TNBC aggressive behaviors could be developed through LIN28/MSI2-YAP1 axis, independently of Let-7 family. We also proposed that inhibition of YAP1/TEAD-mediated transcriptional program may serve as a potential therapeutic strategy for treating a subset of TNBC with LIN28 overexpression.

## Results

### LIN28A expression is prominently upregulated in TNBC tissues and cells

**Fig. 1:**
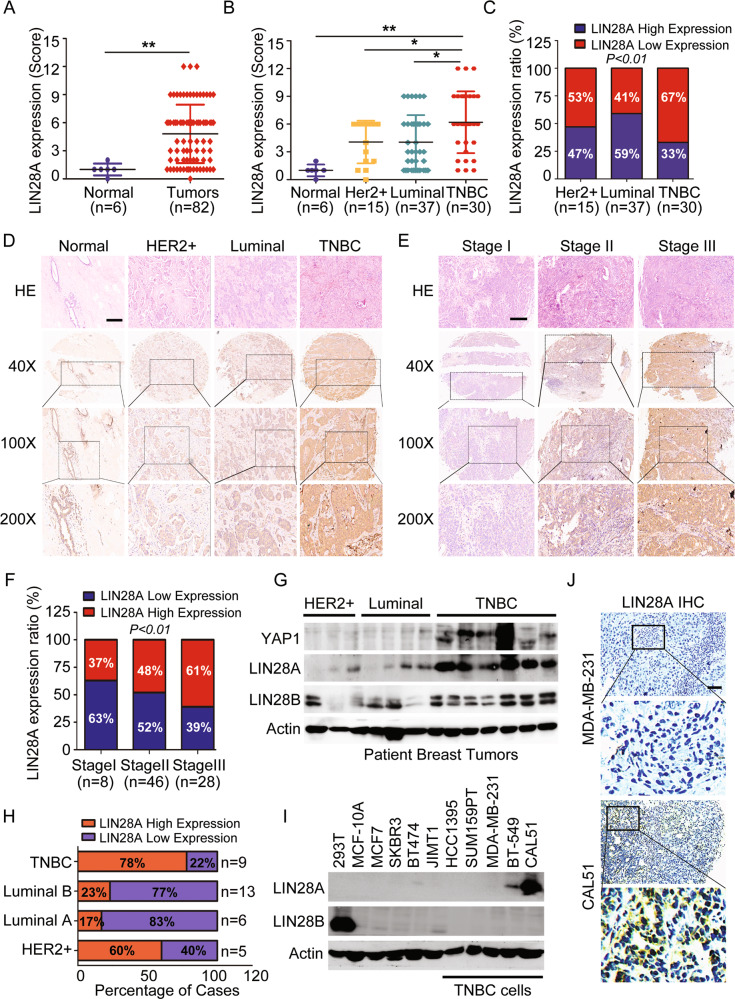
LIN28 expression in human BCs and cells. **A**, **B** LIN28A protein expression levels in 6 normal and 82 BC tissues (with different subtypes) were detected by IHC and evaluated according to the signal scores. IHC staining score ≤ 6 was regarded as low expression and a score > 6 was defined as high expression. **C** Quantitation of LIN28A expression level in 82 BC tissues with different subtypes according to the IHC scores. **D** Representative images indicated the expression of LIN28A in 6 normal tissues and 82 BC tissues detected with IHC. Scale bars: 100 μm. **E** Representative images indicated the expression of LIN28A in 82 BC tissues with different TNM stages detected with IHC. Scale bars: 100 μm. **F** Quantitation of LIN28A expression level in 82 BC tissues with different TNM stages according to the IHC scores. **G** WB analyses of total proteins from patient tumor tissues with different subtypes using the indicated antibodies. **H** Quantitation of LIN28A expression level by WB analyses in different subtypes of BC tissues. **I** WB analyses of total proteins from the 293T cells, MCF-10A cells, and different subtypes of BC cells using the indicated antibodies. **J** IHC analyses of LIN28A protein expression in MDA-MB-231 or CAL51 cell-derived xenograft tissues. Scale bars: 100 μm.

To determine the relevance of LIN28 with BC progression, we first evaluated the expression level of LIN28 in breast tumors, and the correlation between LIN28 expression and progression-free survival of BCs in TCGA-BRCA datasets. The results revealed that LIN28A/B expression was significantly upregulated in primary BCs, and the BC patients with LIN28A/B high expression suffered shorter overall survival (Fig. S1A, B). We then examined LIN28 protein expression level in breast tissue microarrays (TMAs) containing 6 normal adjacent tissues and 82 BC tissues with different subtypes (Fig. S1C–E). We found LIN28A was mainly localized in both cytoplasm and nuclear as previously described, and its expression level was significantly higher in BC tissues based on the IHC score (Fig. 1A). Further characterization of LIN28A expression in TMAs with different BC subtypes revealed that around 50% of the tumor tissues exhibited high LIN28A expression, and its expression ratio with a score > 6 in TNBC was slightly higher compared to other subtypes (Fig. 1B–D). Moreover, we observed that LIN28A expression level was closely associated with the breast TNM stage and showed a higher IHC score in BC tissues at Stage II/III (Fig. 1E, F and Table 1). These data indicated that LIN28A was highly expressed in the BC tissues and may be associated with tumor progression. To validate this conclusion, we further performed the WB analysis using the tissue lysates, including tumor and adjacent normal tissues, collected from 33 patients comprising 9 TNBC and 24 non-TNBC patients. The results showed that LIN28 exhibited a significant higher expression level in TNBC and HER2^+^ BCs, 78% and 60% of these subtypes with LIN28 high expression respectively (Figs. 1G, H, and S1F), which are consistent with previously described in other studies [27, 28]. Subsequently, we also measured LIN28 expression in various BC cells, including the luminal, HER2^+^, and basal subtypes, and found LIN28A, but not LIN28B was highly expressed in TNBC cell lines, such as BT-549 and CAL51 (Figs. 1I and S1G). IHC staining with the xenograft tissues derived from MDA-MB-231 and CAL51 cells further confirmed that LIN28A was prominently expressed in CAL51-derived tumors (Fig. 1J). Taken together, our data indicated that LIN28 was highly expressed in the TNBC tissues and cells, and may be associated with tumor malignancy and poor prognosis.

**Table 1:** Correlation between LIN28A expression and clinicopathological features in breast cancer patients.

Variables	LIN28A				
	Cases	Low	High	Chi-square	*p* Values
Age (y)					
≤55	30	19	11	11.52	0.0007
>55	44	17	27		
Ki67 expression level				20.51	<0.0001
<10%	39	25	14		
≥10%	35	11	24		
TNM Stage				71.29	<0.0001
I	1	1	0		
II	45	24	21		
III	28	11	17		
Subtype				6.926	0.0313
HER2+	14	7	7		
Luminal	33	18	15		
TNBC	27	10	17		

### LIN28 regulates the CSC-like properties, tumor growth, and metastasis

**Fig. 2:**
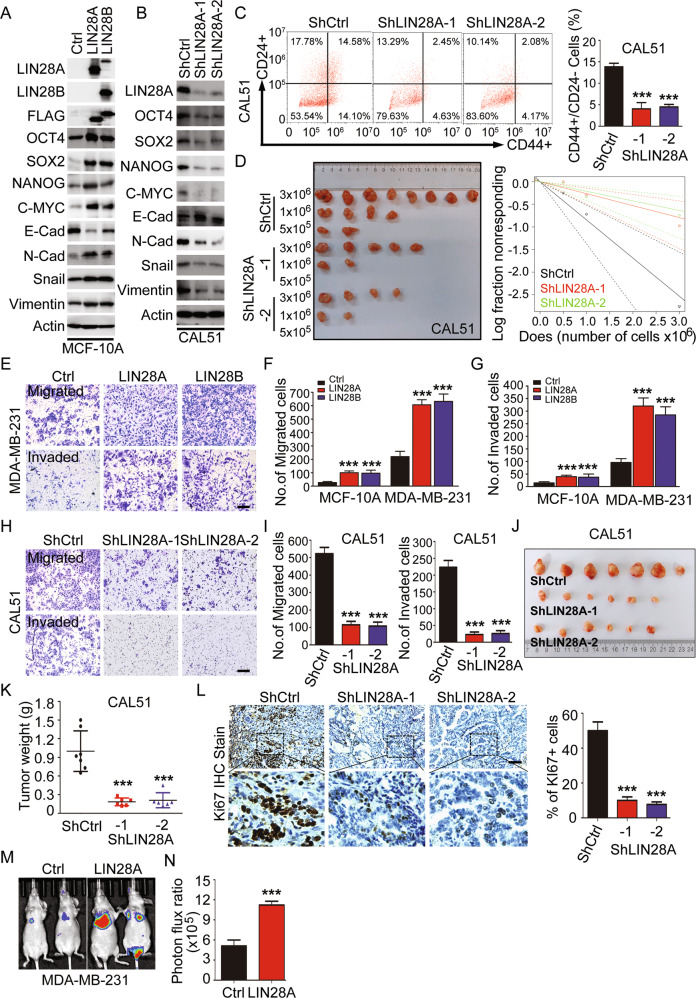
LIN28 regulates CSC-like properties, cell growth and migration/invasion behaviors. **A** WB analyses of total proteins from the MCF-10A cells stably expressing vector control (Ctrl) or Flag-LIN28A/B using the indicated antibodies. **B** WB analyses of total proteins from CAL51 cells stably expressing ShRNA vector control (ShCtrl) or ShLIN28A using the indicated antibodies. **C** Representative images showed the populations of CSCs (CD44^+^/CD24^−/low^) analyzed by flow cytometry in CAL51 cells stably expressing ShCtrl or ShLIN28A. The quantitation data represent means ± SD with 3 biological replicates. **D** Tumor-initiating cell frequency was analyzed by in vivo limiting dilution assay, *n* = 8. **E**–**G** In vitro cell migration/invasion ability was measured in MCF-10A or MDA-MB-231 cells stably expressing Ctrl or Flag-LIN28A/B using the Transwell chamber or Transwell chamber containing the Matrigel as barrier. Representative images of migrated cells were shown. Scale bars: 100 μm. The quantitation data represent means ± SD with 3 biological replicates. **H**–**I** In vitro cell migration/invasion ability was measured in CAL51 cells stably expressing ShCtrl or ShLIN28A using the Transwell chamber or Transwell chamber containing the Matrigel as barrier. Representative images of migrated cells were shown. Scale bars: 100 μm. The quantitation data represent means ± SD with 3 biological replicates. **J**–**K** Xenograft tumor formation assays in NOD-SCID mice using CAL51 cells stably expressing ShCtrl or ShLIN28A. Quantitation of tumor weight represent means ± SD. **L** IHC analyses of Ki67 protein expression in CAL51 cell-derived xenograft tissues stably expressing ShCtrl or ShLIN28A. Scale bars: 100 μm. Quantitation of Ki67+cells represent means ± SD. **M**–**N** Bioluminescence images of lung-colonized tumor cells injected through the tail vein using NOD/SCID mice (*n* = 5 per group), the quantification data were based on the bioluminescence signal intensities.

LIN28 is an important factor for maintaining CSC stemness. To illustrate its regulatory function in TNBC, we firstly performed gain-of-function studies using lentiviruses to stably express LIN28A/B in human mammary epithelial cells MCF-10A or LIN28 negative TNBC cells MDA-MB-231. We found the protein levels of CSC-associated transcription factors (TFs), including OCT4, SOX2, NANOG and C-MYC, were induced upon LIN28 overexpression in MCF-10A. Meanwhile, LIN28 overexpressing cells downregulated the expression of epithelial marker E-cadherin, and upregulated the expressions of mesenchymal markers, including N-cadherin, Snail and Vimentin (Figs. 2A and S2A), demonstrating the critical roles for LIN28 in stemness regulation and the induction of epithelial-mesenchymal transition (EMT) program. Subsequently, loss-of-function studies were performed using lentiviruses expressing two distinct ShRNAs against LIN28A to stably knockdown its expression in TNBC cells. LIN28A downregulation significantly reduced the expressions of CSC-associated TFs and inhibited EMT program (Figs. 2B and S2A). Accumulating evidences have shown that upregulation of CSC-associated TFs and EMT could confer tumor cells with CSC properties [29, 30], while 3D mammosphere formation is a standard assay to assess CSC self-renewal in vitro. We thus analyzed the sphere formation capacities of these cells using this method, and observed LIN28 overexpression dramatically promoted the 3D growth abilities of MCF-10A and MDA-MB-231 cells, while the tumor sphere growth ability was repressed upon downregulation of LIN28A in BT-549 and CAL51 cells (Fig. S2B–D). In addition, CD44^+^/CD24^−/low^ cells were widely used to characterize the CSC population in BCs, we hence evaluated the potential effect of LIN28 on these cell population by flow cytometry analysis. Indeed, the result showed that knockdown of LIN28A expression significantly reduced the percentage of CD44^+^/CD24^−/low^ population in CAL51 cells (Fig. 2C). Limiting dilution assay was considered to be the golden standard for evaluating tumor-initiating frequency in vivo; we then performed this experiment using LIN28A stable knockdown CAL51 cells. The result revealed that LIN28A downregulation significantly decreased tumor incidence (Fig. 2D). All these data demonstrated that LIN28 indeed regulated the CSC-like properties in mammary epithelial and TNBC cells.

Enhanced CSC-like properties are highly associated with tumor growth and metastasis [31]. To determine whether LIN28 regulates the cell growth and cell migration/invasion behaviors, cell growth curve analysis and colony-formation assays were performed to examine the cell proliferation in vitro. Ectopic expression of LIN28 significantly enhanced the epithelial cell proliferation abilities without EGF growth factor, whereas LIN28 downregulation reduced the tumor cell proliferation (Fig. S2E, F). Then we further did the wound healing and transwell assays using LIN28 overexpressing MCF-10A and MDA-MB-231 cells, as well as LIN28 knockdown BT-549 and CAL51 cells to evaluate the cell migration/invasion behaviors in vitro. As anticipated, ectopic expression of LIN28 dramatically enhanced the cell migratory/invasive capabilities (Figs. 2E–G and S2G, H), while knockdown of LIN28A in tumor cells showed an opposite effect (Figs. 2H, I and S2I–L). We further examined the tumorigenicity by tumor xenograft experiments with CAL51 cells stably transfected with ShCtrl or ShLIN28A. Compared with the control cells, tumor cells with LIN28A downregulation caused remarkably reduced tumor growth and size in vivo (Fig. 2J–L). Moreover, to assess the effects of LIN28A on invasive potential in vivo, a xenograft metastasis model was adopted via tail vein injection of MDA-MB-231 cells, and we observed that LIN28A overexpressing cells formed more pulmonary localization than control cells (Fig. 2M, N). Taken together, our results demonstrated that LIN28-enhanced CSC traits could promote tumor cell growth and metastasis in TNBC.

### LIN28 regulates YAP1 activation by inhibition of Hippo pathway

**Fig. 3:**
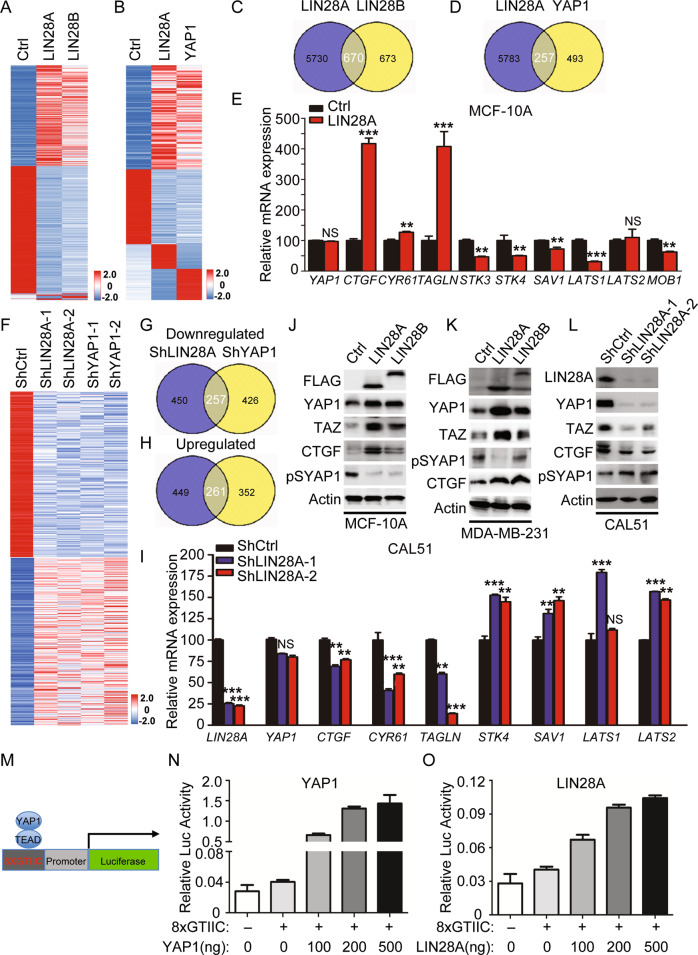
LIN28 induces YAP1 activation and TEAD-mediated transcription output. **A**, **B** The heatmap indicated the gene expression changes induced by overexpression of Flag-LIN28A, 28B, or YAP1 in MCF-10A cells analyzed by RNA-seq. **C**, **D** The commonly regulated genes (up or downregulated) identified by RNA-seq in MCF-10A cells stably overexpressed Flag-LIN28A, 28B, or YAP1 **E** Quantitative real-time PCR to examine the mRNA level of the indicated gene expression in MCF-10A cells stably expressing Ctrl or Flag-LIN28A. The data are shown as the mean ± S.D (*n* = 3). Statistically significant differences are indicated. **F** The heatmap indicated the gene expression changes induced by knockdown of LIN28A or YAP1 in CAL51 cells analyzed by RNA-seq. **G**, **H** The commonly downregulated or upregulated genes identified by RNA-seq in CAL51 cells stably knockdown of LIN28A or YAP1. **I** Quantitative real-time PCR to examine the mRNA level of the indicated gene expression in CAL51 cells stably expressing ShCtrl or ShLIN28A. The data are shown as the mean ± S.D (*n* = 3). Statistically significant differences are indicated. **J**–**L** WB analyses of total proteins from the MCF-10A/MDA-MB-231 cells stably expressing Ctrl or Flag-LIN28A/B, or CAL51 cells stably expressing ShCtrl or ShLIN28A using the indicated antibodies. **M**–**O** Luciferase assay using empty control (pGL3Ctrl) or TEAD-dependent reporter (8XGTIIC) in 293T cells transiently transduced with empty vector, or different concentrations of YAP1 and LIN28A plasmids respectively. The data are shown as the mean ± S.D (*n* = 3).

To elucidate the underlying regulatory mechanism for LIN28A overexpression-induced CSC-like properties, RNA-sequencing was conducted firstly using LIN28A/B overexpressing MCF-10A cells. The analysis of differentially expressed genes (DEGs) between the control and LIN28 overexpressing cells revealed that total 6400 genes were down- or upregulated with more than 1.5-fold changes upon LIN28A overexpression, while 1343 DEGs were identified in LIN28B overexpressing cells. Among them, 670 genes were identified to be common DEGs in LIN28A/B overexpressing MCF-10A cells (Fig. 3A and C), indicating that LIN28A regulated more genes than LIN28B. Further analyzing these common DEGs revealed that YAP1 downstream target genes, including *CYR61* and *CTGF*, were significantly increased upon LIN28A/B overexpression, while the YAP1 upstream kinase-related genes, such as *STK3/4* and *LATS1* were dramatically decreased. However, *YAP1* mRNA level was not affected compared to the control cells (Figs. 3E, S3A and B), indicating that LIN28 may inhibit Hippo signaling and accordingly induce the expression of YAP1 downstream target genes. Then we further performed the DEG analysis with LIN28A and YAP1 overexpressing MCF-10A cells, and identified total 257 genes were commonly regulated, which were around 1/3 of the YAP1-regulated genes (Fig. 3B and D). To validate what we observed, we further conducted the RNA-sequencing using two distinct LIN28A and YAP1 knockdown CAL51 cells. The analysis of DEGs in LIN28A and YAP1 knockdown cells showed that 257 and 261 genes were commonly up- or down-regulated respectively (Fig. 3F–H). qRT-PCR validation revealed that YAP1 downstream genes as mentioned above were downregulated, and YAP1 upstream kinase genes were upregulated in LIN28A knockdown CAL51 cells (Fig. 3I). WB analysis of YAP1 total protein, phosphorylation level at Ser127 and its downstream targets demonstrated that YAP1 was activated in LIN28A overexpressing MCF-10A and MDA-MB-231 cells, while inhibited in LIN28A knockdown CAL51 cells (Figs. 3J–L and S3C). Luciferase assay analysis using the TEAD-dependent reporter (8XGTIIC) revealed that LIN28A/B overexpression could specifically enhance the TEAD-dependent transcriptional activity, as behaved like overexpression of YAP1 in these cells (Figs. 3M–O and S3D). All these data confirmed that LIN28 inhibited Hippo pathway and activated YAP1 in epithelial and TNBC cells.

### YAP1 functions as a key downstream regulator of LIN28A

As the downstream effectors of Hippo pathway, YAP/TAZ were initially identified to be the regulators of cell proliferation and tissue growth [32, 33]. The subsequent researches revealed they also participated in the regulation of CSC-like properties in human BCs [34]. Activation of TAZ was sufficient to endow CSC-like properties to non-CSCs [35], and YAP1 has demonstrated to be recruited by serum response factor to bind the mammary stem cell signature-gene promoters, thereby inducing the stemness-associated genes [36]. Here, we found both of the LIN28 and YAP1 protein levels were increased in CAL51-derived tumor spheres compared in CAL51 monolayer cells (Fig. S3E). In addition, WB and IHC analysis of YAP1 expression in BC tissues, TNBC cells and TNBC cell-derived xenograft tissues validated that YAP1 expression was highly resembled with the expression patterns of LIN28A (Figs. 1G, S1E and S3E–H). Moreover, YAP1 expression in TMAs with different BC subtypes also showed its expression was higher in TNBC subtypes, and its expression level was closely associated with high stage of breast tumors (Fig. S3I–K). These data indicated that YAP1 may play a similar role with LIN28 in TNBC cells. To validate this hypothesis, we performed gain/loss-of-function studies using lentiviruses to stably express YAP1/YAP1S127A in MCF-10A cells, and YAP1 ShRNAs in CAL51 cells. As behaved like LIN28 ectopically-expressed cells, YAP1 overexpression/activation upregulated the protein levels of CSC-associated TFs and promoted the EMT program in MCF-10A cells, whereas YAP1 knockdown suppressed these protein expressions and showed the opposite effects in CAL51 cells (Figs. S4A and 4C). Further evaluation of CSC-related characteristics with these cells revealed that MCF-10A cells with YAP1 activation resulted in an increase in number of mammosphere, while CAL51 cells infected with ShYAP1 displayed strikingly decreased mammosphere numbers and percentages of CD44^+^/CD24^−/low^ population (Figs. S4B and 4D, E). Cell growth curve analysis and colony-formation assay showed that YAP1 activation dramatically enhanced MCF-10A cell proliferation ability without EGF, while cell proliferation rate was reduced upon downregulation of YAP1 in CAL51 cells (Fig. S4G–J). Wound healing assay also showed that downregulation of YAP1 reduced the CAL51 cell migration ability in vitro (Fig. S4K, L). Taken together, all these findings demonstrated that YAP1 indeed played a similar role with LIN28 in TNBC cells.

**Fig. 4:**
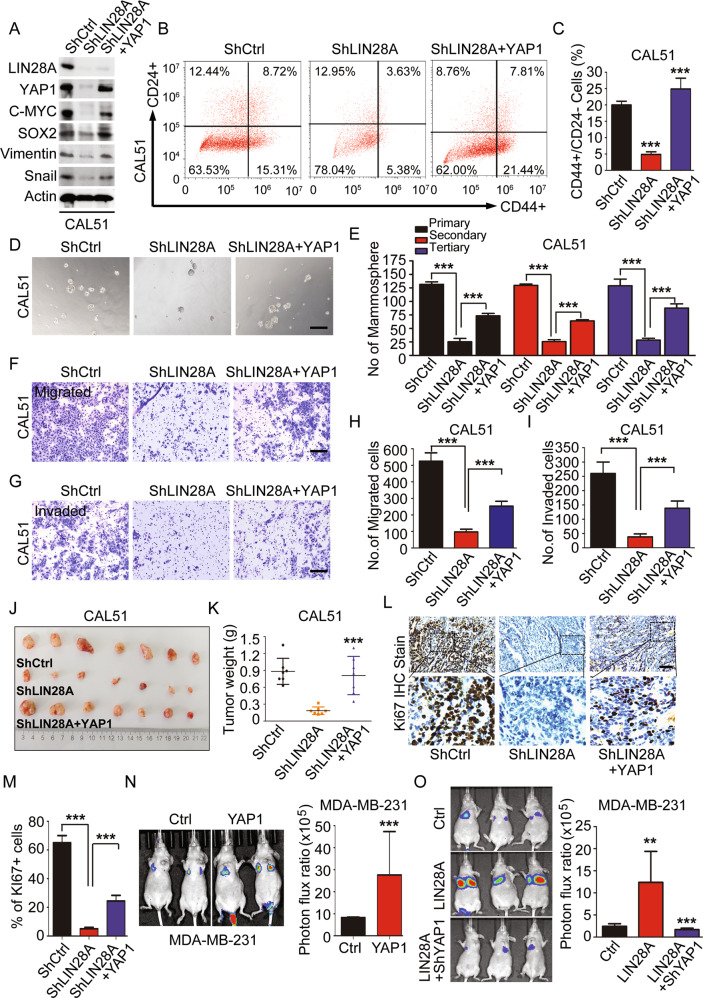
YAP1 functions as a key downstream regulator of LIN28. **A** WB analyses of total proteins from CAL51 cells stably expressing ShCtrl, ShLIN28A, or ShLIN28A+Flag-YAP1 using the indicated antibodies. **B**, **C** Representative images showed the populations of CSCs (CD44^+^/CD24^−/low^) analyzed by flow cytometry in CAL51 cells stably expressing ShCtrl, ShLIN28A, or ShLIN28A+Flag-YAP1. The quantitation data represent means ± SD with 3 biological replicates. **D**, **E** Tumorsphere formation was analyzed in CAL51 cells stably expressing ShCtrl, ShLIN28A, or ShLIN28A+Flag-YAP1. Representative images of tumorspheres are shown. Scale bars: 100 μm. The quantitation data represent means ± SD with 3 biological replicates. **F**–**I** In vitro cell migration/invasion ability was measured in CAL51 cells stably expressing ShCtrl, ShLIN28A, or ShLIN28A+Flag-YAP1 using the Transwell chamber or Transwell chamber containing the Matrigel as barrier. Representative images of migrated cells are shown. Scale bars: 100 μm. The quantitation data represent means ± SD with 3 biological replicates. **J**, **K** Xenograft tumor formation assays in NOD-SCID mice using CAL51 cells stably expressing ShCtrl, ShLIN28A, or ShLIN28A+Flag-YAP1. Quantitation of tumor weight represents means ± SD. **L**, **M** IHC analyses of Ki67 protein expression in CAL51 cell-derived xenograft tissues stably expressing ShCtrl, ShLIN28A, or ShLIN28A+Flag-YAP1. Scale bars: 100μm. Quantitation of Ki67+ cells represents means ± SD. **N**, **O** Bioluminescence images of lung-colonized tumor cells injected through the tail vein using NOD/SCID mice (*n* = 5 per group), the quantification data are based on the bioluminescence signal intensities.

Then to clarify whether YAP1 activation in LIN28 overexpressing cells is linked to the phenotypes we observed above, we rescued the YAP1 expression in LIN28A stably knockdown CAL51 cells using lentivirus. We observed that overexpression of YAP1 in LIN28A knockdown CAL51 cells could rescue the expressions of CSC-associated TFs and EMT-associated markers (Fig. 4A). Correspondingly, the number of the tumor sphere and percentage of CD44^+^/CD24^−/low^ population were significantly increased in LIN28A knockdown CAL51 cells followed by YAP1 overexpression (Fig. 4B–E). These results indicated that YAP1 functioned as a downstream effector of LIN28A to mediate the CSC-associated properties in TNBC cells. Subsequently, we further analyzed the effect of YAP1 on LIN28A-regulated tumorigenesis and cell migration/invasion phenotypes in vitro and in vivo, and found rescue of YAP1 expression in LIN28A knockdown CAL51 cells could potentially improve the tumor growth of xenografts, and the cell migration/invasion abilities caused by LIN28A downregulation (Figs. 4F–M and S4M, N). Moreover, YAP1 activation prominently enhanced, while knockdown of YAP1 in LIN28A overexpressing MDA-MB-231 cells dramatically repressed their capacities of pulmonary localization via the xenograft metastasis model (Fig. 4N, O). Collectively, all these findings demonstrated that YAP1 functioned as a key downstream regulator of LIN28A-mediated CSC properties, tumorigenesis and metastasis.

### The regulation of YAP1 by LIN28 is independent of Let-7

**Fig. 5:**
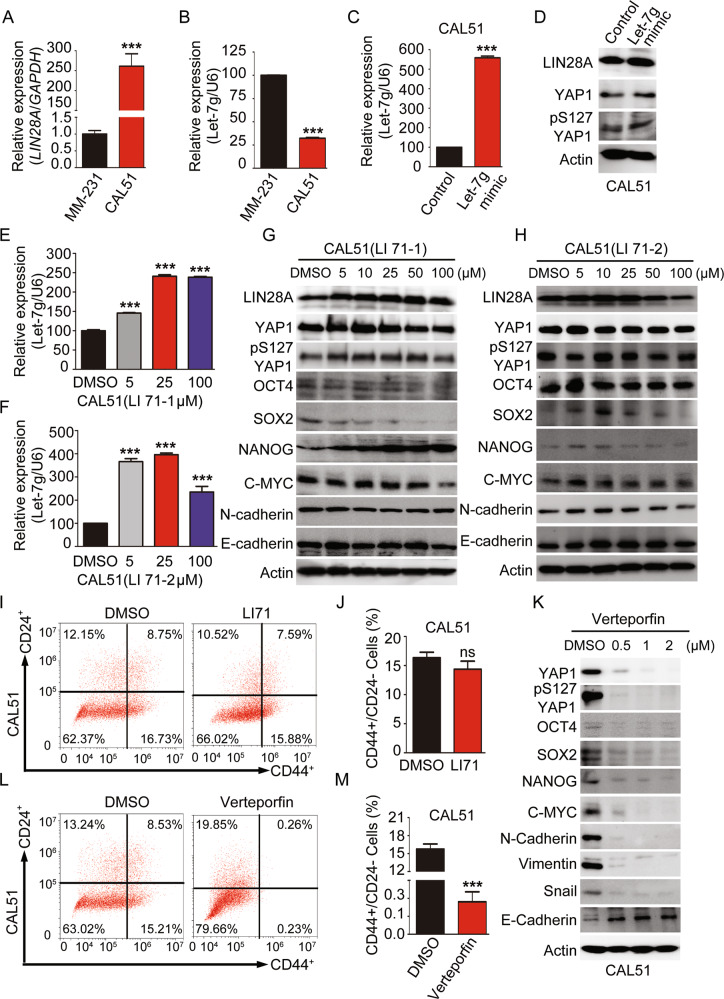
LIN28-induced YAP1 activation is independent of Let-7. **A**, **B** Quantitative real-time PCR to examine the mRNA level of the indicated gene expression in MDA-MB-231 and CAL51 cells. The data are shown as the mean ± S.D (*n* = 3). **C**, **D** Quantitative real-time PCR and WB analyses of Let-7g and YAP1 activation level in CAL51 cells transiently transfected with control and Let-7g mimic. The data are shown as the mean ± S.D (*n* = 3). **E**, **F** Quantitative real-time PCR analyses of Let-7g expression level in CAL51 cells treated with different concentrations of LIN28/Let-7 inhibitor for 48 h. The data are shown as the mean ± S.D (*n* = 3). **G**, **H** WB analyses of total proteins from CAL51 cells treated with different concentrations of LIN28/Let-7 inhibitors for 48 h using the indicated antibodies. **I**, **J** Representative images showed the populations of CSCs (CD44^+^/CD24^−/low^) analyzed by flow cytometry in CAL51 cells treated with DMSO or LI71. The quantitation data represent means ± SD with 3 biological replicates. **K** WB analyses of total proteins from CAL51 cells treated with different concentrations of YAP1/TEAD inhibitor for 48 h using the indicated antibodies. **L**, **M** Representative images showed the populations of CSCs (CD44^+^/CD24^−/low^) analyzed by flow cytometry in CAL51 cells treated with DMSO or Verteporfin. The quantitation data represent means ± SD with 3 biological replicates.

Let-7 family has been identified as an essential downstream target of LIN28 in many cancers. To determine whether YAP1 activation by LIN28 is dependent of Let-7, we firstly analyzed the expression of Let-7g in TNBC cells, which is the most relevant Let-7 isoform with tumor metastasis and poor patient survival in BCs. We observed Let-7g expression was negatively correlated with LIN28A, and dramatically repressed in CAL51 cells (Fig. 5A, B). Then CAL51 cells transiently transfected with either let-7g mimic or control were used to analyze the potential regulation effect of Let-7g on YAP1. The WB data showed that forced expression of let-7g has no effect on either YAP1 expression or activation (Fig. 5C, D). Moreover, we also treated the CAL51 cells with different concentrations of LI71 (inhibitor for LIN28/Let-7 interactions), which has been demonstrated to inhibit LIN28’s activity on let-7 miRNAs in cancer and embryonic stem cells (ESCs) [21, 37]. We observed that increase of the LI71 treatment concentrations (LI71–1/2 represents two different chirality) significantly induced the Let-7g expression in CAL51 cells (Fig. 5E, F). However, release of the Let-7 inhibition from LIN28A did not affect the protein levels of YAP1 and LIN28A, as well as EMT-associated markers. Despite that SOX2 protein level was gradually decreased with the increase of LI71 concentrations, most of the other CSC-associated TFs were almost even in this process (Fig. 5G, H). These data demonstrated the activation of YAP1 by LIN28A was independent of Let-7. Further analysis of the CSC-associated properties with LI71-treated CAL51 cells revealed that inhibition of LIN28-Let-7 interactions did not affect the percentages of CD44^+^/CD24^−/low^ population, tumorsphere growth, as well as the in vitro cell migration/invasion abilities (Figs. 5I, J and S5A–F). However, when we treated CAL51 cells with different concentrations of Verteporfin, we found both YAP1 total protein and phosphorylation levels were dramatically reduced. Meanwhile, the expressions of CSC-associated TFs and EMT markers were significantly decreased (Fig. 5K). Further analysis of the CSC-associated properties with Verteporfin-treated CAL51 cells revealed that inhibition of YAP1-TEAD interactions, rather than LIN28-Let-7 activity, played a more essential role for LIN28-induced phenotypes in TNBC cells (Figs. 5L, M and S5G–L). Taken together, all these findings indicated that LIN28-YAP1 axis-induced CSC-like properties were independent of Let-7 in TNBC.

### LIN28 recruits MSI2 to inhibit Hippo pathway

**Fig. 6:**
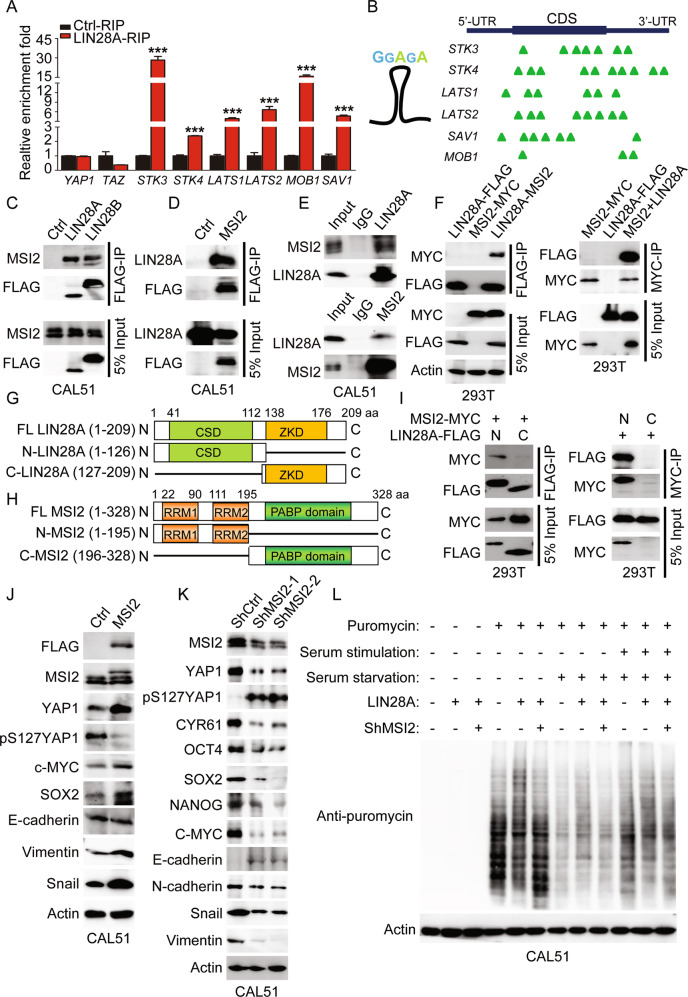
LIN28 recruits MSI2 to regulate YAP1 activation. **A** RNA immunoprecipitated (RIP) with anti-Flag antibody followed by qRT-PCR to analyze LIN28 binding of *YAP1* and *TAZ* mRNAs, and other indicated target mRNAs in MCF-10A cells stably expressing Ctrl or Flag-LIN28A. The enrichment folds of mRNAs present in the LIN28 IP are relative to the control IP. The experiment was done in 3 biological replicates and data from a representative experiment are shown. **B** Consensus motifs within LIN28 clusters identified in ESCs and 293T cells, and the mapped GGAGA motifs in indicated genes are shown with green triangle. **C** WB analyses of total proteins (Input) or anti-Flag antibody immunoprecipitated (Flag-IP) proteins from CAL51 cells stably expressing vector control (Ctrl) or Flag-LIN28A/B using the indicated antibodies. **D** WB analyses of Input or Flag-IP proteins from CAL51 cells stably expressing Ctrl or Flag-MSI2 using the indicated antibodies. **E**, **F** Co-IP showing the interaction between endogenous LIN28A and MSI2 in CAL51 cells, or exogenous LIN28A and MSI2 in 293T cells. **G**, **H** Schematic of full-length LIN28A and MSI2 protein domains and their truncated constructs. **I** Co-IP analysis to determine the regions of LIN28A required for interacting with MSI2, and the regions of MSI2 required for interacting with LIN28A. **J**, **K** WB analyses of total proteins from CAL51 cells stably transfected with Ctrl, Flag-MSI2, or ShCtrl, ShMSI2-1, and ShMSI2-2 using the indicated antibodies. **L** WB analyses of puromycin-labeled nascent proteins in CAL51 cells stably expressing the indicated vectors. Puromycin (10 μg/ml) was added to the culture medium of CAL51 cells 30 min before harvest (with and without serum starvation), Actin was detected as an internal control.

As a RNA-binding protein, previous studies have reported that LIN28 could function as either a transcriptional or translational regulator to directly enhance the target mRNA translation [10, 23–26]. Given our above observation that LIN28-mediated YAP1 activation is independent of Let-7, we want to test whether LIN28 could directly bind to mRNAs of *YAP1*/*TAZ* or their upstream kinases. To this end, RNA immunoprecipitation (RIP) assay was performed in LIN28 overexpressing MCF-10A cells, and the followed qPCR analysis showed that LIN28 directly bound to the mRNAs of *STK3/4*, *LATS1/2*, *SAV1* and *MOB1*, but not *YAP1*/*TAZ* (Figs. 6A and S6A, B). Consistent with these observations, we indeed identified lots of conserved GGAGA motifs previously reported in LIN28 expressing cells, were enriched in LIN28-binding target mRNAs, in particular within exons and 3-UTR (Fig. 6B). Since the mRNA levels of YAP1/TAZ upstream kinases were downregulated in LIN28 overexpressing MCF-10A cells based on our RNA-seq data (Figs. 3E and S3A, B), we speculate that there must be other factors interacting with LIN28 to mediate its inhibitory effect on Hippo pathway. We thus performed a LIN28-interacting protein screening in MCF-10A cells based on previously published mass spectrophotometry (MS) data (Fig. S6C) [10, 38–40]. All these known candidates were mainly implicated in diverse gene regulatory functions, including mRNA binding and metabolism (hnRNP A1 and PABPC4), translation regulation (EEF1G, EIF3B, DHX9 and DDX3), and other RNA-binding proteins (MSI1/2). Here we have done the screening adhering to three principles, the candidates should be (1) interacted with LIN28 and validated by coimmunoprecipitation (Co-IP) assay, (2) highly expressed in LIN28-positive TNBC cells, and (3) associated with Hippo pathway inhibition and YAP1 activation. We have tested all these candidates and found MSI2 was specifically interacted with LIN28 in both MCF-10A and CAL51 cells via Co-IP analysis, which was also shown for endogenous LIN28A and MSI2 (Fig. 6C–E). Co-IP of ectopically expressed FLAG-tagged LIN28A or MYC-tagged MSI2 from 293T cells also validated the interaction between LIN28A and MSI2 (Fig. 6F). A series of truncated LIN28A or MSI2 constructs were generated and transfected into 293T cells, and Co-IP assays showed that the N-terminal regions of both LIN28A (CSD domain) and MSI2 (RRM domain) were required for their interactions (Fig. 6G–I). Besides, WB and IHC analyses of MSI2 expression in TNBC cells and xenograft tissues showed it was prominently expressed in BT-549 and CAL51 cells (Fig. S6D, E). Moreover, overexpression of MSI2 in CAL51 cells dramatically induced YAP1 total protein and reduced its phosphorylation at Ser127. Meanwhile, CSC-associated TFs were increased and EMT program was enhanced upon MSI2 upregulation, while knockdown of MSI2 in CAL51 cells showed totally opposite effects (Fig. 6J, K). Functionally, we found knockdown of MSI2 in CAL51 cells dramatically reduced the tumor sphere growth, cell migration/invasion abilities (Fig. S6F–K). More importantly, we found MSI2 expression level was remarkably increased in primary BCs, and its high expression was associated with the shorter overall survival of patients (Fig. S6L, M). Further analysis of the expression of MSI2 and LIN28 in BC tissues showed a positive correlation (Fig. S6N). All these findings supported our hypothesis that LIN28-MSI2 synergistically enhanced CSC-like properties in TNBC by inactivation of Hippo pathway.

### LIN28A/MSI2-YAP1 axis induces tumor growth and metastasis in xenograft model and human BCs

**Fig. 7:**
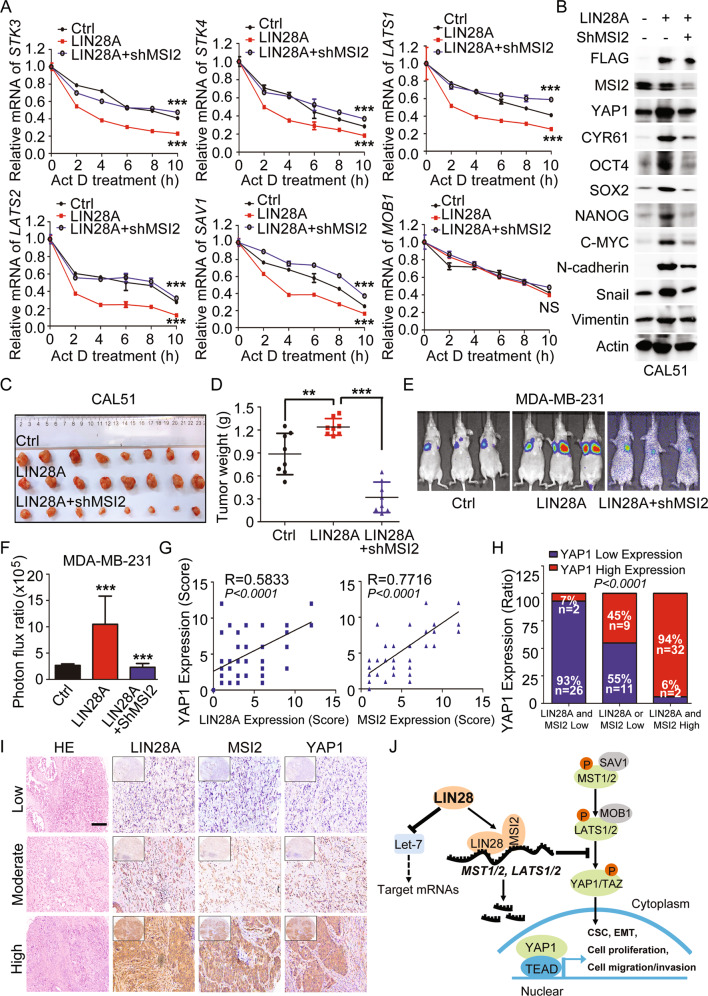
LIN28A/MSI2-YAP1 axis induces tumor growth and metastasis in xenograft model and human BCs. **A** RNA stability curves plotted using qPCR expression values versus time. ANCOVA analysis was used in determining statistical significance. **B** WB analyses of total proteins from CAL51 cells stably expressing Ctrl, Flag-LIN28A or Flag-LIN28A + ShMSI2 using the indicated antibodies. **C**, **D** Xenograft tumor formation assays in NOD-SCID mice using CAL51 cells stably expressing Ctrl, LIN28A, or LIN28A + ShMSI2. Quantitation of tumor weight represent means ± SD. **E**, **F** Bioluminescence images of lung-colonized tumor cells injected through the tail vein using NOD/SCID mice (*n* = 5 per group), the quantification data are based on the bioluminescence signal intensities. **G** Positive correlation of LIN28A or MSI2 with YAP1 expression was assessed using Pearson correlation coefficient analysis according to IHC scores. **H, I** Representative images and quantitation of YAP1 expression level in 82 BC tissues with different levels of LIN28A or MSI2 expression according to the IHC scores. Scale bars: 100 μm. **J** Schematic of the proposed model to illustrate that LIN28/MSI2-YAP1 regulatory axis induces the CSC properties, tumor growth, and metastasis in TNBC via inactivation of Hippo pathway independently of Let-7.

MSI2, one of the two members of Musashi RNA-binding family, is participated in the post-transcriptional regulation via controlling the translation efficiency or target mRNA stability in many cancers. Like in BCs, MSI2 was found to promote the aggressive phenotypes by binding the mRNA of estrogen receptor *ESR1*, and thereby increasing its RNA stability and translation efficiency [41]. To this end, protein translation efficiency was measured in LIN28A overexpressing or LIN28A+ShMSI2 CAL51 cells by puromycin incorporation assay. We saw that either LIN28A overexpression or knockdown of MSI2 in CAL51 cells did not show obvious effect on the global protein synthesis efficiency (Fig. 6L). Then to test whether LIN28/MSI2-mediated Hippo pathway inhibition is dependent on the regulation of target mRNA stability, RNA decay curves for the YAP1 upstream kinase genes were assayed using an Actinomycin D time course in Ctrl, LIN28A overexpressing or LIN28A+ShMSI2 CAL51 cells followed by qPCR analyses. As shown in Fig. 7A, LIN28A overexpressing cells dramatically induced the target mRNA decay, including *MST1/2*, *LATS1/2* and *SAV1*, while these target mRNAs were significantly more stable in the absence of MSI2, demonstrating that LIN28-mediated mRNA decay was dependent on MSI2 recruitment. We then further analyzed the YAP1 activation level in LIN28A overexpressing CAL51 cells stably transduced with MSI2 ShRNA. As anticipated, knockdown of MSI2 in LIN28A overexpressing CAL51 cells dramatically attenuated the LIN28A-induced YAP1 expression and activation, as well as the expressions of CSC and EMT-associated markers (Fig. 7B). Subsequently, the tumorsphere formation and transwell assays also showed MSI2 downregulation in LIN28A overexpressing cells could repress the tumorsphere growth, as well as the cell migration/invasion abilities caused by LIN28A overexpression (Fig. S7A–F). More importantly, in vivo xenograft assays also revealed that LIN28A-enhanced tumor growth and pulmonary localization were dependent on LIN28/MSI2-mediated YAP1 activation (Fig. 7C–F and S7G). Taken together, our results unraveled that LIN28 inhibited Hippo pathway via recruiting MSI2 and de-stabilizing the mRNA transcript of YAP1 upstream kinases in TNBC cells, thereby enhancing CSC-associated properties, tumor cell growth and metastasis.

To determine the potential association of LIN28/MSI2-mediated YAP1 activation with BC patients, breast TMAs we previously used for LIN28A expression analysis were used for MSI2 and YAP1 IHC staining. As observed like LIN28A and YAP1, MSI2 also showed higher expression level in BC tissues compared in normal tissues, and its expression level was significantly associated with a TNM stage (Fig. S7H–I). Moreover, BC patients with high levels of both LIN28A and MSI2 had a higher level of YAP1 expression (32/34), whereas those with low levels of LIN28A and MSI2 had a lower level of YAP1 expression (26/28), indicating a correlated expression of LIN28A/MSI2 and YAP1 (Fig. 7G–I). In addition, co-expressions of LIN28A/MSI2 and YAP1 in BCs were dramatically associated with the TNM stage (Fig. S7J, K). Collectively, these results highlighted the clinical relevance of LIN28/MSI2-mediated YAP1 regulation in BC patients, and targeting of YAP/TEAD-mediated transcriptional output may represent a treatment strategy for LIN28/MSI2-overexpressing BCs.

## Discussion

In this study, we demonstrate that LIN28 negatively regulates the Hippo pathway by complexing with MSI2, leading to the activation of YAP1, which in turn enhances CSC-like properties, tumorigenesis, and metastasis in TNBC cells. More importantly, the regulation of Hippo pathway by LIN28 in this process is independent of Let-7 (Fig. 7J). Our findings thus unravel a novel LIN28/MSI2-YAP1 regulatory axis to induce the CSC properties, tumor growth and metastasis, which may serve as a potential therapeutic strategy for the treatment of a subset of TNBC with LIN28 overexpression.

LIN28-mediated stem cell maintenance, including pluripotent stem cells and CSCs, plays essential roles for both embryonic development and tumorigenesis. However, the downstream targets of LIN28 are obscured. In our previous study, we have identified Yap1 as a crucial downstream effector of Lin28 and demonstrated Yap1/Tead-mediated transcriptional output was responsible for Lin28-mediated glial cell differentiation [42]. Here, we further showed that YAP1 was a key downstream target for LIN28-enhanced CSC-like properties, tumorigenesis and metastasis in TNBC cells. As the downstream effector of Hippo pathway, YAP1 is regulated by the kinase cascade MST1/2 and LATS1/2. LATS-dependent phosphorylation restrains YAP1 stability, nuclear localization, and transcriptional activity [43]. Functionally, YAP1 was initially identified to be a size controller of multiple organs [43–45]. Emerging evidences revealed that YAP1 also took the center stage in cancers, by which to regulate the CSC self-renewal and tumor cell behaviors [46–48]. In BC cells, YAP1 has been found to occupy the gene promoters of mammary stem cell signature to induce breast CSC properties, and it was highly enriched in breast CSCs with CD44^+^/CD24^−/low^ phenotype [35, 36]. Here, we further demonstrated YAP1 functioned as a key downstream regulator for LIN28-regulated CSC properties in TNBC cells.

As a RNA-binding protein, LIN28 was widely regarded to function through inhibiting the maturation of let-7 family. In our study, we found that inhibition of LIN28/Let-7 interactions was not responsible for LIN28-induced YAP1 activation, as well as the enhanced CSC-like properties in TNBC cells, suggesting this process is independent of Let-7, and some other mechanisms may be adopted by LIN28 to regulate YAP1. Emerging evidences have revealed that LIN28 could either function as a DNA-based regulator by recruiting other epigenetic modifiers, like Tet1 in ESCs [26], or directly affect target mRNA metabolism and translation. Through our RNA-seq and qRT-PCR analyses, we did not observe the YAP1 mRNA changes in MCF-10A or TNBC cells with LIN28 gain/loss-of-function, suggesting LIN28 did not affect YAP1 transcription. Instead of that, we found LIN28 could directly bind the mRNAs of YAP1 upstream kinases, including *STK3/4*, *LATS1/2*, and repress their stability. Consistent with these observations, we did see YAP1 phosphorylation level at Ser127 was decreased upon LIN28 upregulation, which lead to increasing YAP1 nuclear localization and protein stability. All these data supported that LIN28-mediated YAP1 regulation was dependent of canonical Hippo pathway. Moreover, combining with previously-published MS and our Co-IP data, we identified another RNA-binding protein MSI2, could be recruited by LIN28 to bind the mRNAs of YAP1 upstream kinases. MSI2 is emerging as a regulator of multiple critical biological processes relevant to cancer initiation, progression, and drug resistance through maintaining CSC populations, including BCs [49, 50]. In pancreatic ductal adenocarcinoma, recent study has revealed that MSI2-mediated tumorigenesis was associated with Hippo pathway [51]. Here, we further revealed that MSI2 functioned as a negative regulator of Hippo pathway via interacting with LIN28. In summary, our study provided the mechanistic insights that LIN28-MSI2 regulated the CSC-like properties in TNBC cells via modulating Hippo pathway and independently of Let-7, thereby promoting tumorigenesis and metastasis. Therefore, inhibition of YAP1/TEAD-dependent transcriptional output may serve as a potential treatment strategy for the TNBC with LIN28 overexpression.

## Materials and methods

### Cell lines and cell culture

Human mammary epithelial cells and BC cell lines were obtained from American Type Culture Collection (ATCC). MDA-MB-231, BT-549, CAL51, and 293T cells were grown in DMEM medium (Gibco, USA) supplemented with 10% heat-inactivated fetal bovine serum (FBS) (Gibco, USA) and 2 mmol/L L-glutamine. MCF-10A cell was cultured in DMEM/F12 medium (Gibco, USA) containing additional 5% horse serum, 10 μg/mL insulin, 20 ng/mL epidermal growth factor (EGF), 250 ng/μL hydrocortisone and 100 ng/ml Cholera toxin. All the cell culture mediums were added with 100 U/mL penicillin/streptomycin and all the cells were maintained under standard cell culture conditions with 5% CO_2_ at 37 °C.

### DNA constructs, Reverse-transcription, Real-time PCR and Western blot

DNA constructs: the lentiviral expression constructs pUbi-MCS-3xFlag (GV358), subcloned with human *LIN28A/B*, *MSI2*, *YAP1* or *YAP1S127A* were purchased from the GeneChem company https://www.genechem.com.cn/. pLKO.1-Puro subcloned with human *LIN28A*, *MSI2,* or *YAP1* ShRNAs were purchased from TranSheepBio company http://www.transheep.com/. For lentiviral production and infection, lentiviral plasmid (1.2 μg), including either the overexpressing or ShRNA plasmids, together with 0.8 μg of packaging plasmid pSPAX2 (Addgene #12260) and 0.5 μg of envelope expressing plasmid (Addgene #12259) were transiently co-transfected into the 293T cells using the Lipofectamine 2000 reagent according to the manufacturer’s instructions. 48 h after transfection, the lentivirus supernatant was collected and filtered with 0.45 μm membrane filters (Millipore). Human cells were infected in the presence of 5 μg/mL polybrene and selected with 1 μg/mL puromycin for 72 h. The oligo sequences of human *LIN28A*, *MSI2* or *YAP1* ShRNAs were listed in Supplementary Table 1, the empty vector control (GV358) and ShRNA control vectors (pLKO.1-Puro) were used as Ctrl and ShCtrl in above-mentioned lentiviral transfection experiments.

#### Reverse-transcription and real-time PCR

Total RNAs were isolated from epithelial cells or BC cells using Trizol reagent (Invitrogen, Shanghai). The RNA was reverse transcribed to cDNA using the PrimeScript RT reagent Kit (TaKaRa) according to manufacturer’s protocol. Expression level of *GAPDH* was used as an internal control. Real-time PCR was performed using the SYBR Premix Ex Taq (TaKaRa) on Bio-Rad CFX96 Real-Time Thermocycler (CFX96, Bio-Rad Laboratories, Hercules, CA) machine. The sequences of primers used in this study were listed in Supplementary Table 1.

#### Western blot (WB)

Total cells were lysed using the RIPA buffer (Beyotime) containing the cocktail of protease inhibitors and phosphatase inhibitors for 30 min on ice. For subcellular distribution analysis assay, nuclear and cytoplasmic fractions were isolated following the instructions (Gentex). The cytoplasmic proteins were extracted using the harvest buffer A (10 mM HEPES (pH = 7.9), 50 mM NaCl, 0.1 mM EDTA, 1 mM PMSF, 2 mg/ml Pepstatin A and freshly added 1 mM DTT, 10 mM NaF,17.5 mM β-glycerophosphate, 0.5 M sucrose, 0.5%Triton X-100). The nuclear extractions were extracted using the Buffer B (10 mM HEPES (pH = 7.9), 500 mM NaCl, 0.1 mM EDTA, 1 mM PMSF, 2 mg/ml Pepstatin A and freshly added 1 mM DTT, 0.1 mM EGTA, 0.1%NP-40). The cell lysates were then resolved on 10% SDS-PAGE and electrotransferred to PVDF membranes (Millipore). The membranes were blocked with 5% BSA for 1 h and immunoblotted with the respective primary antibodies overnight at 4 °C. The primary and secondary antibodies and corresponding dilution ratios used in this study were listed in Supplementary Table 2.

### Co-IP, RIP, Luciferase assays, RNA stability analysis, and puromycin incorporation assay

#### Co-IP

Flag-IP was performed as previously described [52]. Briefly, the human cells were lysed in 50 mM Tris-HCl, pH7.5, 100 mM NaCl, 1% Triton X-100, 0.1 mM EDTA, 0.5 mM MgCl_2_, 10% glycerol, protease inhibitor cocktail (Roche), phosphatase inhibitor cocktail (Roche), and 10 μM pervanadate (NEB). Lysates were incubated with Anti-FLAG M2 Magnetic Beads (Sigma, M8823) overnight at 4 °C. Antibody/protein complexes were washed with lysis buffer for four times and analyzed by WB.

#### RIP

RNA-binding protein immunoprecipitation (RIP) assays were performed using the EZ-Magna RIP^TM^ Kit (No.17-701; Millipore) in accordance with the manufacturer’s instructions. In brief, around 2 × 10^7^ cells were lysed in RIP lysis buffer provided in the Kit, subsequently, the lysates were incubated with Anti-Flag M2 magnetic beads (No.8223; Sigma) at 4 °C for overnight, and then the precipitate complexes were washed with washing buffer for six times and treated with proteinase K. Immunoprecipitated RNA was extracted using the phenol/chloroform method, and the RNA was subjected to reverse transcription and qPCR analysis. qPCR primers were listed in Supplementary Table 1.

#### Luciferase assay

Luciferase assay was performed as previously described [52]. Briefly, Renilla plasmid (5 ng per well) was used as the internal transfection control, whereas the pGL3 empty vector (100 ng per well) was used as the experimental control, 8XGTIIC plasmid was used to detect the TEAD-dependent transcriptional output. Lipofectamine 2000 (Invitrogen) was used to conduct transfection experiments following the manufacturer’s instructions. 48 h after transfection, luciferase activity was detected using a Dual-Luciferase Reporter Assay System (Promega, E1910) according to the manufacturer’s instructions. The average values of the tested constructs were normalized to the activity of the pGL3 empty construct and the Renilla activity.

#### RNA stability analysis

CAL51 cells stably overexpressed LIN28A or LIN28A OE+ShMSI2 were seeded into 6-well plates for overnight, and then the cells were exposed to Actinomycin D (5 μg/mL) for 0, 2, 4, 6, 8, 10 h respectively. The cells were then harvested for total RNA isolation and Reverse-transcription. Finally, the mRNA levels of *STK3*, *STK4*, *LATS1*, *LATS2*, *MOB1*, *and SAV1* were analyzed by qPCR.

#### Puromycin incorporation assay

CAL51 cells stably overexpressed LIN28A or LIN28A OE+ShMSI2 were seeded at 2.5 × 10^5^ cells/well in 6-well plates overnight prior to serum starvation. For serum stimulation, cells were maintained in the media supplemented with 10% fetal bovine serum for an optimized period (2 h for CAL51 cell before adding the puromycin). Subsequently, 10 μg/ml of puromycin stock was added into the cell cultures and the cells were incubated for 30 min at 37 °C. Finally, the cells were collected with ice-cold RIPA lysis buffer, and 10 μg of the whole cell lysates were used to do WB and assess the puromycin integration efficiency.

### Flow cytometry analysis

Firstly, the BC cells were trypsinized, harvested and washed with PBS buffer, and then 2 × 10^5^ single-cell suspensions were incubated in 400 μL running buffer with anti-human CD44 (FITC-conjugated, Cat #: 555478) and anti-human CD24 (PE-conjugated, Cat #: 555428) at room temperature for 30 min. Finally, the percentages of labeled cells were analyzed using the CytoFLEX LX flow cytometer (Beckman).

### Immunohistochemistry (IHC) staining assay

IHC staining: paraffin-embedded tissues were sectioned, deparaffinized and rehydrated through an alcohol series, followed by antigen retrieval with sodium citrate buffer, and then 3% H_2_O_2_ was used to block endogenous peroxidase activity for 10 min. Subsequently, the slides were blocked using 10% goat serum at room temperature for 1 h, and then incubated with appropriate primary antibodies at 4 °C overnight. The next day, IHC samples were used for secondary antibody incubations for 1 h at room temperature, and followed by detection with DAB. Stained sections were observed and captured using the microscope.

### Cell proliferation, colony-formation, wound healing, tumor spheroid growth, migration and invasion assays

#### Cell proliferation assay

The human cells were seeded in a 24-well plate overnight at seeding density of 5000 cells/well, the medium was changed every 2 days. The cells from three independent wells were digested for trypan blue staining and calculated continuously for seven days to draw the growth cure and analyze using GraphPad Prism 5 software.

#### Colony-formation assay

The human cells were seeded in a 6-well plate at seeding density of 1000 cells per well on day 1 and the medium was changed every 2 days. The colonies were fixed with 4% paraformaldehyde on day 14 and then stained with 0.5% crystal violet before counting by inverted phase contrast microscope.

#### Wound healing assay

The human cells were seeded in a 6-well plate and let the cells grown until 80%−90% confluence. A sterile 200 μL pipette tip was used to make a wound on the cell monolayer and the fragments were washed with PBS, and serum-free medium was added. The wound gap was visualized and photographed under the inverted microscope at different time points.

#### Mammary sphere growth assay

The human cells were trypsinized and seeded in an ultralow-attachment 24-well plate (Corning) with 5000 cells per well in serum-free DMEM/F12 medium, supplemented with 10 ng/mL bFGF, 20 ng/mL EGF, 0.4% BSA, 4 μg/ml insulin and B27 supplement (diluted 1:50). The cells were cultivated at 37 °C with 5% CO_2_ for 7 days. The mammospheres were collected by centrifugation and re-seeded for the secondary and tertiary of mammosphere formation respectively. After each replating, the sphere numbers (cells ≥ 50) were calculated under the microscope on day 7.

#### Cell migration and invasion assays

Firstly, the human cells were harvested and replated with a serum-free culture medium. For cell migration assay, 1 × 10^4^ viable cells in 200 μL serum-free medium were plated in the upper chamber with the non-coated membrane with 8 µm pore size. For cell invasion assay, 2 × 10^4^ viable cells were seeded in top chambers that pre-coated with Matrigel (BD Biosciences, CA). The medium supplemented with serum was used as a chemical attractant in the lower chamber. The cells were incubated for 48 h and the migration cells on the undersurface of the membrane were fixed with 4% paraformaldehyde and stained with 0.5% crystal violet. Three fields were randomly selected to take photograph and counted for statistical analysis using an inverted microscope.

### Xenograft assay in nude mice

Animal research was conducted in the guidance of an approved for the Institutional Animal Care and Use Committee of Sun Yat-Sen University. 4–6 weeks old female BALB/c nude mice were obtained from the GemPharmatech Co., Ltd. (Jiangsu). CAL51 cells were harvested and 3 × 10^6^ cells were injected subcutaneously on the flank of each BALB/c nude mouse. Tumors can be observed about 3–4 weeks after subcutaneous inoculation. The mice were euthanized when the xenografts reached 1000 mm [20] in tumor volume and the Xenograft tumors were collected, weighed and picture taken. The maximum (a) and minimum (b) length of tumor was measured with a calipers and tumor volume was calculated using the following formula: a^2^ × b × 0.4. Lung metastasis model: MDA-MB-231 cells stably overexpressed Luciferase gene (around 1 × 10^6^ per mouse in 200 μl PBS) were intravenously introduced into the female BALB/c nude mice (4–6 weeks old). Bioluminescence signals in mice were monitored using Xenogen IVIS Spectrum and the data were analyzed with the Living Image software (Caliper Life Sciences). When the Bioluminescence signals reached to 1 × 10^7^ and the mouse was euthanized. Lung tissues were harvested for taking picture and H&E staining. Limiting dilution assay: CAL51 cells stably infected with ShCtrl or ShLIN28A lentivirus were diluted into different cell densities and injected subcutaneously on the flank of each BALB/c nude mouse. Then the tumorigenesis rate of each group was measured. The frequency of CSCs was calculated by publically available ELDA website (http://bioinf.wehi.edu.au/software/elda/).

### Human samples and tissue microarray

Human BC specimens used in this study for WB analysis were collected from the Seventh Affiliated Hospital of Sun Yat-sen University. All patients received written informed consent and all the collections of the BC samples were approved by each hospital committee. The study was authorized by the Ethics Committee of Sun Yat-sen University.

### RNA-sequencing

Total mRNA was isolated from BC cells using Trizol reagent (Invitrogen). The RNA-seq libraries were constructed and sequenced utilizing the VAHTSTM Stranded mRNA-seq Library Prep Kit for Illumina v2 (Vazyme Biotech, cat. no NR612) according to the manufacturer’s instructions. And then the sequencing was analyzed using by Illumina NovaSeq 6000 platform PE150.

### Statistical analysis

All the in vitro experiments were carried out at least three times, and the data were indicated as mean ± standard deviation (SD). Studies involving comparison between the two groups were analyzed by an unpaired Student’s t-test or among multiple groups were compared first with by one-way ANOVA with post hoc comparison by the Tukey’s test as indicated by GraphPad Prism 5 software. P-value is indicated by asterisks in the Figures (*P* < 0.05 [*]; *P* < 0.01 [**] and *P* < 0.001 [***]). Differences of *P* < 0.05 and lower were considered statistically significant.

## Supplementary information

Supplementary figure and table legends

Supplementary figure 1

Supplementary figure 2

Supplementary figure 3

Supplementary figure 4

Supplementary figure 5

Supplementary figure 6

Supplementary figure 7

Supplementary table 1

Supplementary table 2
