# Supplementary material for: RNA-binding protein complex LIN28/MSI2 enhances cancer stem cell-like properties by modulating Hippo-YAP1 signaling and independently of Let-7
Source: Oncogene. 2022 Jan 31;41(11):1657–72. doi: 10.1038/s41388-022-02198-w (PMC8913359; doi:10.1038/s41388-022-02198-w)
Supplement: Supplementary file 1 — Supplementary figure and table legends [file 41388_2022_2198_MOESM1_ESM.docx]

**Supplementary figure legends**

**Figure S1. LIN28 expression in human breast cancers and cells.**

(A). LIN28A/B mRNA expression levels in The Cancer Genome Atlas (TCGA) breast cancer RNA-seq dataset.

(B). Kaplan-Meier analysis of overall survival curves for breast cancer patients that were grouped according to LIN28A/B expression level.

(C). The whole slide scans for HE and LIN28A IHC stain with 6 normal tissues and 74 breast cancer tissues.

(D). IHC analyses of LIN28A protein expression in human breast tumor tissues (#1: stage II, #2-8: stage I).

(E). Representative images indicated the low (score ≤ 6) and high (score >6) expression level of LIN28A in breast cancer tissues detected with IHC. Scale bars: 100μm.

(F). Western blot analyses of total proteins from TNBC patient tissues using the indicated antibodies. A: adjacent normal tissues; T: tumors.

(G). qRT-PCR analysis of LIN28A mRNA level in MCF-10A cells and different subtypes of breast cells.

**Figure S2. LIN28** **regulates CSC-like properties, cell growth and migration/invasion behaviors.**

(A). Western blot analyses of total proteins from MDA-MB-231 or BT-549 cells stably expressing Ctrl and Flag-LIN28A/B, or ShCtrl and ShLIN28A respectively using the indicated antibodies.

(B-D). Tumorsphere formation was analyzed in MCF-10A/MDA-MB-231 cells stably expressing Ctrl or Flag-LIN28A/B and in CAL51/BT-549 cells stably expressing ShCtrl or ShLIN28A. Representative images of tumorspheres were shown. Scale bars: 100μm. The quantitation data represent means ± SD with 3 biological replicates.

(E). Colony-formation and cell growth curve assays were performed to analyze the cell proliferation in MCF-10A cells stably expressing Ctrl or Flag-LIN28A/B without EGF. The quantitation data represent means ± SD with 3 biological replicates.

(F). Colony-formation and cell growth curve assays were performed to analyze the cell proliferation in CAL51 cells stably expressing ShCtrl or ShLIN28A. The quantitation data represent means ± SD with 3 biological replicates.

(G-H). In vitro cell migration ability was measured in MDA-MB-231 cells stably expressing Ctrl or Flag-LIN28A/B by wound healing assay. Representative images at different time points were shown. Scale bars: 100μm. The quantitation data represent means ± SD with 3 biological replicates.

(I-J). In vitro cell migration ability was measured in CAL51 cells stably expressing ShCtrl or ShLIN28A by wound healing assay. Representative images at different time points were shown. Scale bars: 100μm. The quantitation data represent means ± SD with 3 biological replicates.

(K-L). In vitro cell invasion ability was measured in BT-549 cells stably expressing ShCtrl or ShLIN28A using the Transwell chamber containing the Matrigel as barrier. Representative images of migrated cells were shown. Scale bars: 100μm. The quantitation data represent means ± SD with 3 biological replicates.

**Figure S3. LIN28 induces YAP1 activation and TEAD-mediated transcription output.**

(A). The heatmap indicated the gene expression changes induced by overexpression of Flag-LIN28A in MCF-10A cells analyzed by RNA-seq.

(B). Quantitative real-time PCR to examine the mRNA level of the indicated gene expression in MCF-10A cells stably expressing Ctrl or Flag-LIN28B. The data are shown as the mean ± S.D (n=3). Statistically significant differences were indicated.

(C). Western blot analyses of nuclear and cytoplasmic fractions from MDA-MB-231 cells stably expressing Ctrl or Flag-LIN28A/B.

(D). Luciferase assay using empty control (pGL3Ctrl) or TEAD-dependent reporter (8XGTIIC) in 293T cells transiently transduced empty vector, or different concentrations of YAP1 and LIN28B plasmid respectively. The data are shown as the mean ± S.D (n=3).

(E). Western blot analyses of proteins from CAL51 cells and CAL51 cell-derived tumor spheres using the indicated antibodies.

(F). Quantitation of YAP1 expression level by western blot analyses in different subtypes of breast cancer tissues.

(G). Western blot analyses of proteins from TNBC cells using the indicated antibodies.

(H). IHC analyses of YAP1 protein expression in MDA-MB-231 or CAL51 cell-derived xenograft tissues.

(I). YAP1 protein expression levels in 6 normal and 82 breast cancer tissues (with different subtypes) were detected by IHC according to the score method above.

(J). Quantitation of YAP1 expression level in 82 breast cancer tissues with different subtypes and stages according to the IHC score.

(K). IHC analyses of YAP1 protein expression in human breast tumor tissues (#1: stage II, #2-8: stage I).

**Figure S4. YAP1 functions as a key downstream regulator of LIN28A.**

(A). Western blot analyses of total proteins from MCF-10A cells stably expressing vector control (Ctrl), Flag-YAP1 or Flag-YAP1S127A using the indicated antibodies.

(B). Tumorsphere formation was analyzed in MCF-10A cells stably expressing Ctrl, Flag-YAP1, Flag-YAP1S127A. Representative images of spheres were shown. Scale bars: 100μm. The quantitation data represent means ± SD with 3 biological replicates.

(C). Western blot analyses of total proteins from CAL51 cells stably expressing ShRNA vector control (ShCtrl) or ShYAP1 using the indicated antibodies.

(D). Representative images showed the populations of CSCs (CD44^+^/CD24^-/low^) analyzed by flow cytometry in CAL51 cells stably expressing ShCtrl or ShYAP1. The quantitation data represent means ± SD with 3 biological replicates.

(E-F). Tumorsphere formation was analyzed in CAL51 cells stably expressing ShCtrl or ShYAP1. Representative images of spheres were shown. Scale bars: 100μm. The quantitation data represent means ± SD with 3 biological replicates.

(G-H). Colony-formation and cell growth curve assays were performed to analyze the cell proliferation in MCF-10A cells stably expressing Ctrl, Flag-YAP1, Flag-YAP1S127A.

(I-J). Colony-formation and cell growth curve assays were performed to analyze the cell proliferation in CAL51 cells stably expressing ShCtrl or ShYAP1.

(K-L). In vitro cell migration ability was measured in CAL51 cells stably expressing ShCtrl or ShYAP1 by wound healing assay. Representative images at different time points were shown. Scale bars: 100μm. The quantitation data represent means ± SD with 3 biological replicates.

(M-N). In vitro cell migration ability was measured in CAL51 cells stably expressing ShCtrl, ShLIN28A or ShLIN28A+Flag-YAP1 by wound healing assay. Representative images at different time points were shown. Scale bars: 100μm. The quantitation data represent means ± SD with 3 biological replicates.

**Figure S5. LIN28-induced YAP1 activation is independent of Let-7.**

(A-B). Tumorsphere formation was analyzed in CAL51 cells treated with DMSO or 0.5μM LI71 and representative images of tumorspheres were shown. Scale bars: 100μm. The quantitation data represent means ± SD with 3 biological replicates.

(C-D). In vitro cell migration ability was measured in CAL51 cells treated with DMSO or 0.5μM LI71 using the Transwell chamber. Representative images of migrated cells were shown. Scale bars: 100μm. The quantitation data represent means ± SD with 3 biological replicates.

(E-F). In vitro cell invasion ability was measured in CAL51 cells treated with DMSO or 0.5μM LI71 using the Transwell chamber containing the Matrigel as barrier. Representative images of invaded cells were shown. Scale bars: 100μm. The quantitation data represent means ± SD with 3 biological replicates.

(G-H). Tumorsphere formation was analyzed in CAL51 cells treated with DMSO or 0.5μM Verteporfin and representative images of tumorspheres were shown. Scale bars: 100μm. The quantitation data represent means ± SD with 3 biological replicates.

(I-J). In vitro cell migration ability was measured in CAL51 cells treated with DMSO or 0.5μM Verteporfin using the Transwell chamber. Representative images of migrated cells were shown. Scale bars: 100μm. The quantitation data represent means ± SD with 3 biological replicates.

(K-L). In vitro cell invasion ability was measured in CAL51 cells treated with DMSO or 0.5μM Verteporfin using the Transwell chamber containing the Matrigel as barrier. Representative images of invaded cells were shown. Scale bars: 100μm. The quantitation data represent means ± SD with 3 biological replicates.

**Figure S6. LIN28 recruits MSI2 to regulate YAP1 activation.**

(A-B). RNA immunoprecipitated (RIP) with anti-Flag antibody followed by qRT-PCR to analyze LIN28 binding of indicated genes in MCF-10A cells stably expressing Ctrl or Flag-LIN28B. The enrichment folds of mRNAs present in the LIN28 IP are relative to the control IP. The experiment was done in 3 biological replicates and data from a representative experiment are shown.

(C). Western blot analyses of total proteins (Input) or anti-Flag antibody immunoprecipitated (Flag-IP) proteins from MCF-10A cells stably expressing vector control (Ctrl) or Flag-LIN28A/B using the indicated antibodies.

(D). Western blot analyses of proteins from TNBC cells using the indicated antibodies.

(E). IHC analyses of MSI2 protein expression in CAL51 cell-derived xenograft tissues.

(F-G). Tumorsphere formation was analyzed in CAL51 stably expressing ShCtrl or ShMSI2. Representative images of tumorspheres were shown. Scale bars: 100μm. The quantitation data represent means ± SD with 3 biological replicates.

(H-K). In vitro cell migration/invasion ability was measured in CAL51 cells stably expressing ShCtrl or ShMSI2 using the Transwell chamber or Transwell chamber containing the Matrigel as barrier. Representative images of migrated cells were shown. Scale bars: 100μm. The quantitation data represent means ± SD with 3 biological replicates.

(L). MSI2 mRNA expression levels in The Cancer Genome Atlas (TCGA) breast cancer RNA-seq dataset including 114 normal and 1097 tumor tissues.

(M). Kaplan-Meier analysis of overall survival curves for Breast cancer patients that were grouped according to MSI2 expression level.

(N). Positive correlation of MSI2 with LIN28A expression was assessed using Pearson correlation coefficient analysis.

**Figure 7. LIN28 regulated-CSC properties, cell growth and migration/invasion behaviors are dependent of MSI2 recruitment.**

(A-B). Tumor sphere formation was analyzed in CAL51 cells stably expressing Ctrl, Flag-LIN28A or Flag-LIN28A+ShMSI2 and representative images of tumorspheres were shown. Scale bars: 100μm. The quantitation data represent means ± SD with 3 biological replicates.

(C-D). In vitro cell migration ability was measured in CAL51 cells stably expressing Ctrl, Flag-LIN28A or Flag-LIN28A+ShMSI2 using the Transwell chamber. Representative images of migrated cells were shown. Scale bars: 100μm. The quantitation data represent means ± SD with 3 biological replicates.

(E-F). In vitro cell invasion ability was measured in CAL51 cells stably expressing Ctrl, Flag-LIN28A or Flag-LIN28A+ShMSI2 using the Transwell chamber containing the Matrigel as barrier. Representative images of invaded cells were shown. Scale bars: 100μm. The quantitation data represent means ± SD with 3 biological replicates.

(G). IHC analyses of Ki67 protein expression in CAL51 cell-derived xenograft tissues stably expressing Ctrl, LIN28A or LIN28A+ShMSI2. Scale bars: 100μm.

(H). MSI2 protein expression levels in 6 normal and 74 breast cancer tissues (with different subtypes) were detected by IHC according to the score method above.

(I). Quantitation of MSI2 expression level in 74 breast cancer tissues with different subtypes and stages according to the IHC score.

(J). Representative images indicated the expression of LIN28A, MSI2 and YAP1 in 74 breast cancer tissues with different stage I-III detected with IHC. Scale bars: 100μm.

(K). IHC analyses of MSI2 protein expression in human breast tumor tissues (#1: stage II, #2-8: stage I).

**Supplementary table legends**

Supplementary table 1: Oligo sequences used in this paper.

Supplementary table 2: Antibody list used in this paper.
