# Supplementary figures and images for: RNA-binding protein complex LIN28/MSI2 enhances cancer stem cell-like properties by modulating Hippo-YAP1 signaling and independently of Let-7
Source: Oncogene. 2022 Jan 31;41(11):1657–72. doi: 10.1038/s41388-022-02198-w (PMC8913359; doi:10.1038/s41388-022-02198-w)

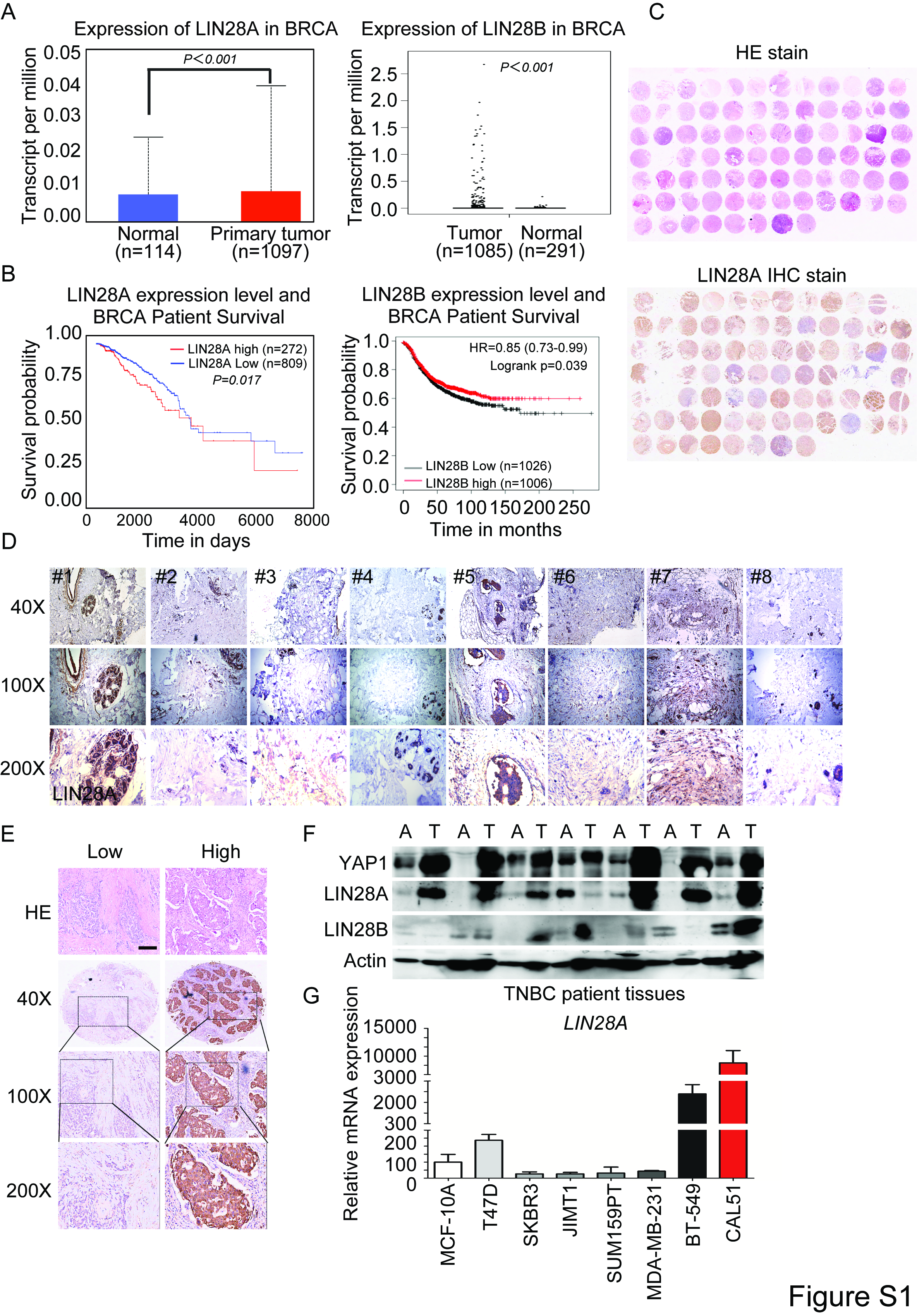

Supplement: Supplementary file 2 — Supplementary figure 1 [file 41388_2022_2198_MOESM2_ESM.tif]

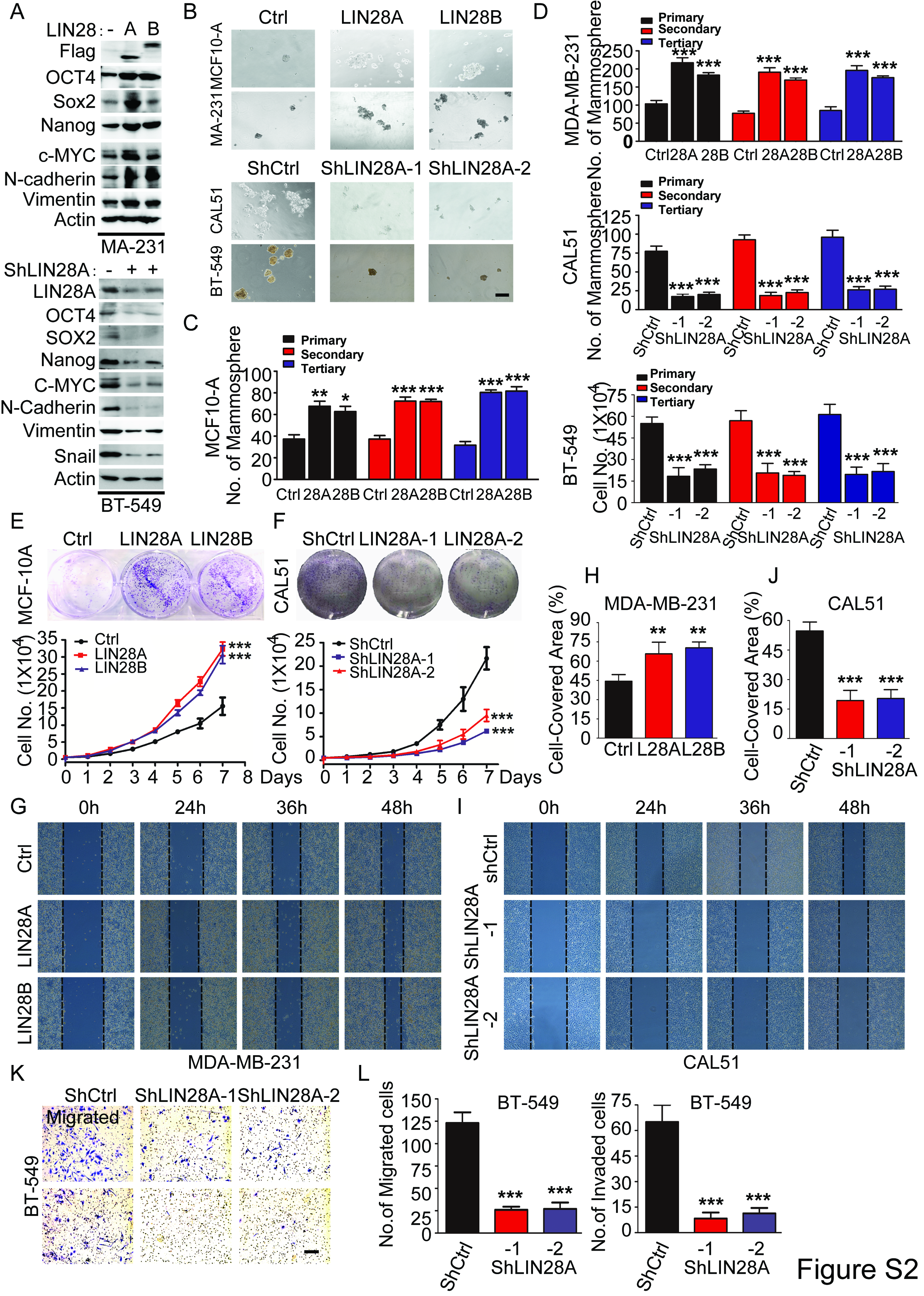

Supplement: Supplementary file 3 — Supplementary figure 2 [file 41388_2022_2198_MOESM3_ESM.tif]

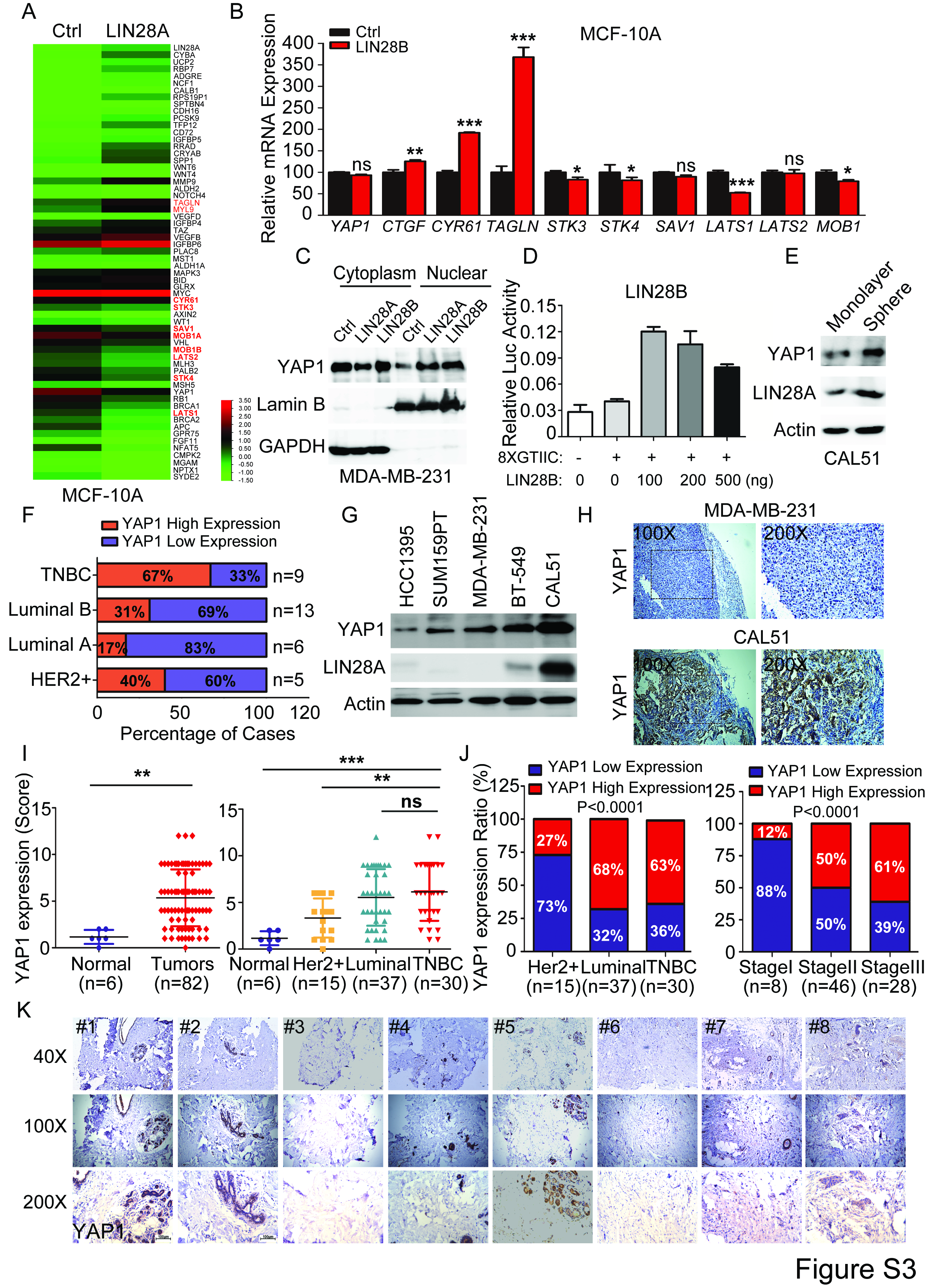

Supplement: Supplementary file 4 — Supplementary figure 3 [file 41388_2022_2198_MOESM4_ESM.tif]

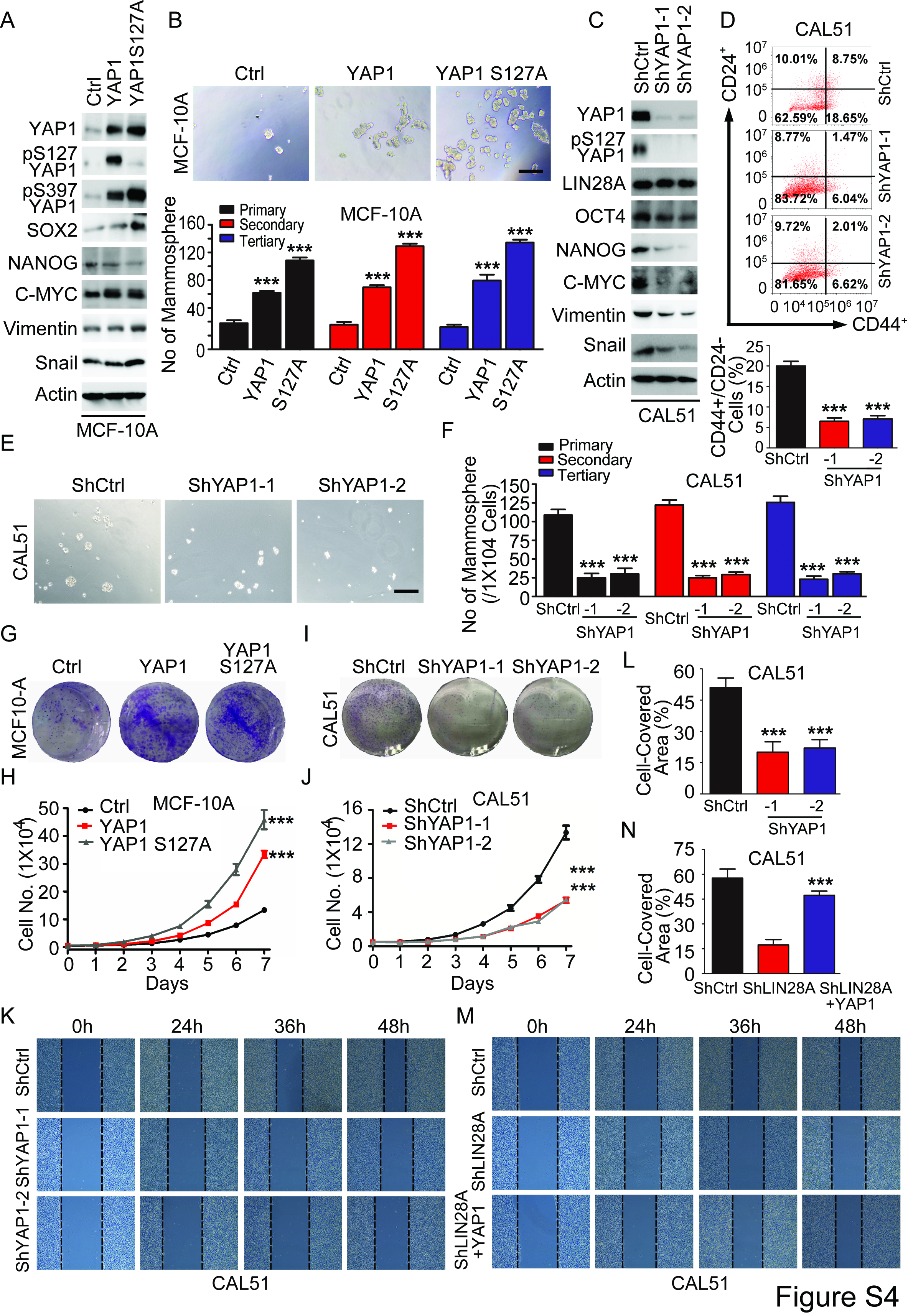

Supplement: Supplementary file 5 — Supplementary figure 4 [file 41388_2022_2198_MOESM5_ESM.tif]

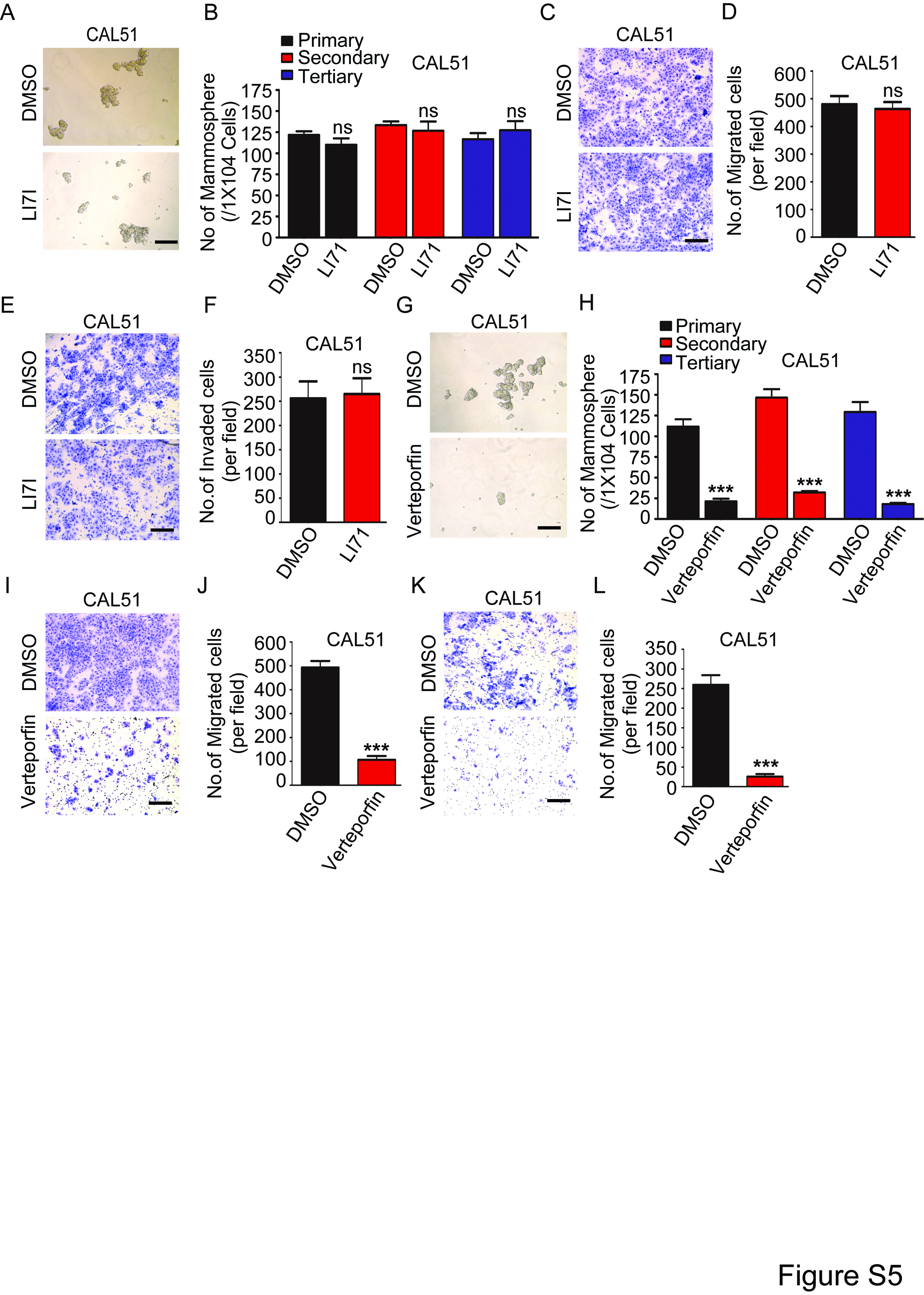

Supplement: Supplementary file 6 — Supplementary figure 5 [file 41388_2022_2198_MOESM6_ESM.tif]

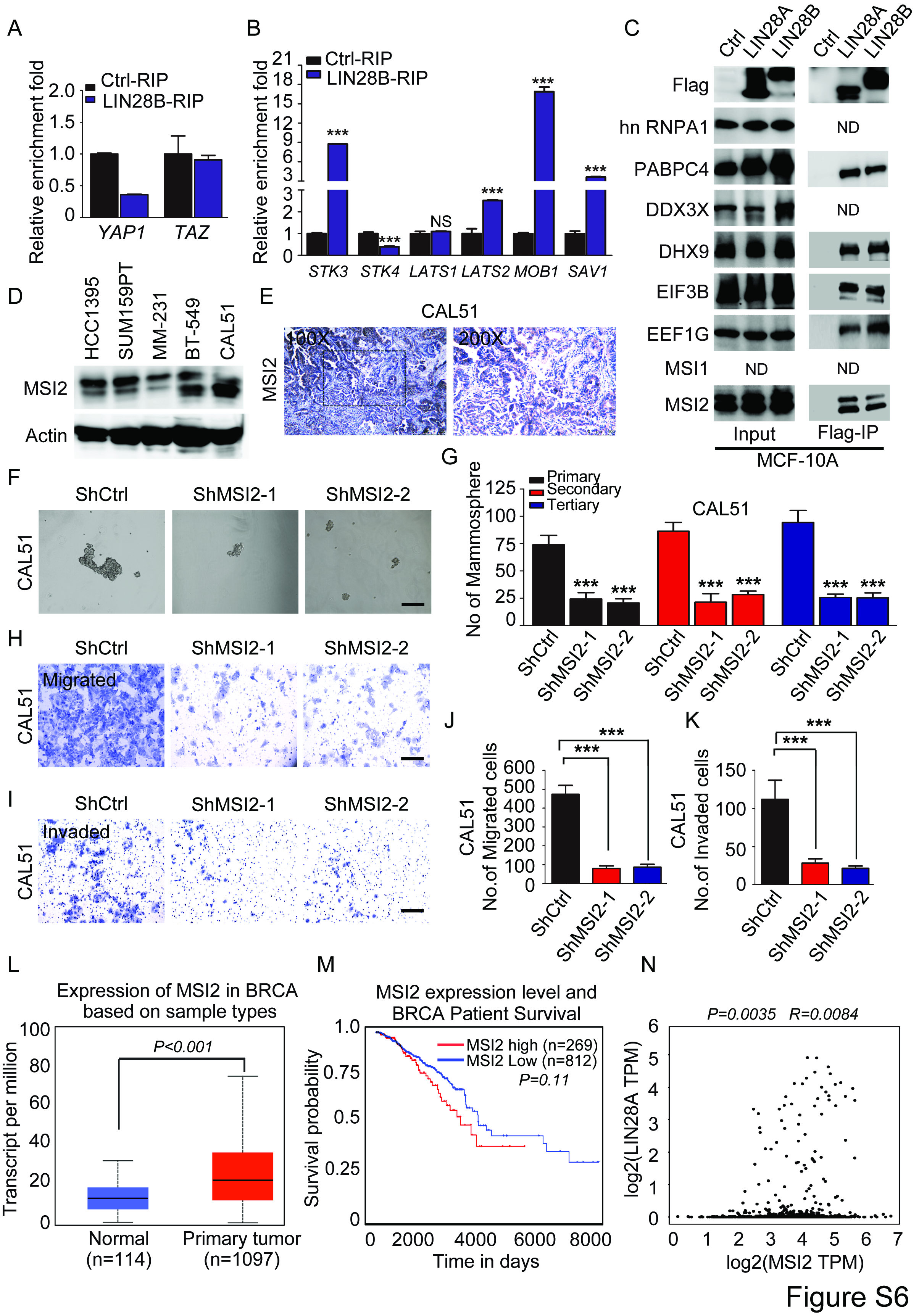

Supplement: Supplementary file 7 — Supplementary figure 6 [file 41388_2022_2198_MOESM7_ESM.tif]

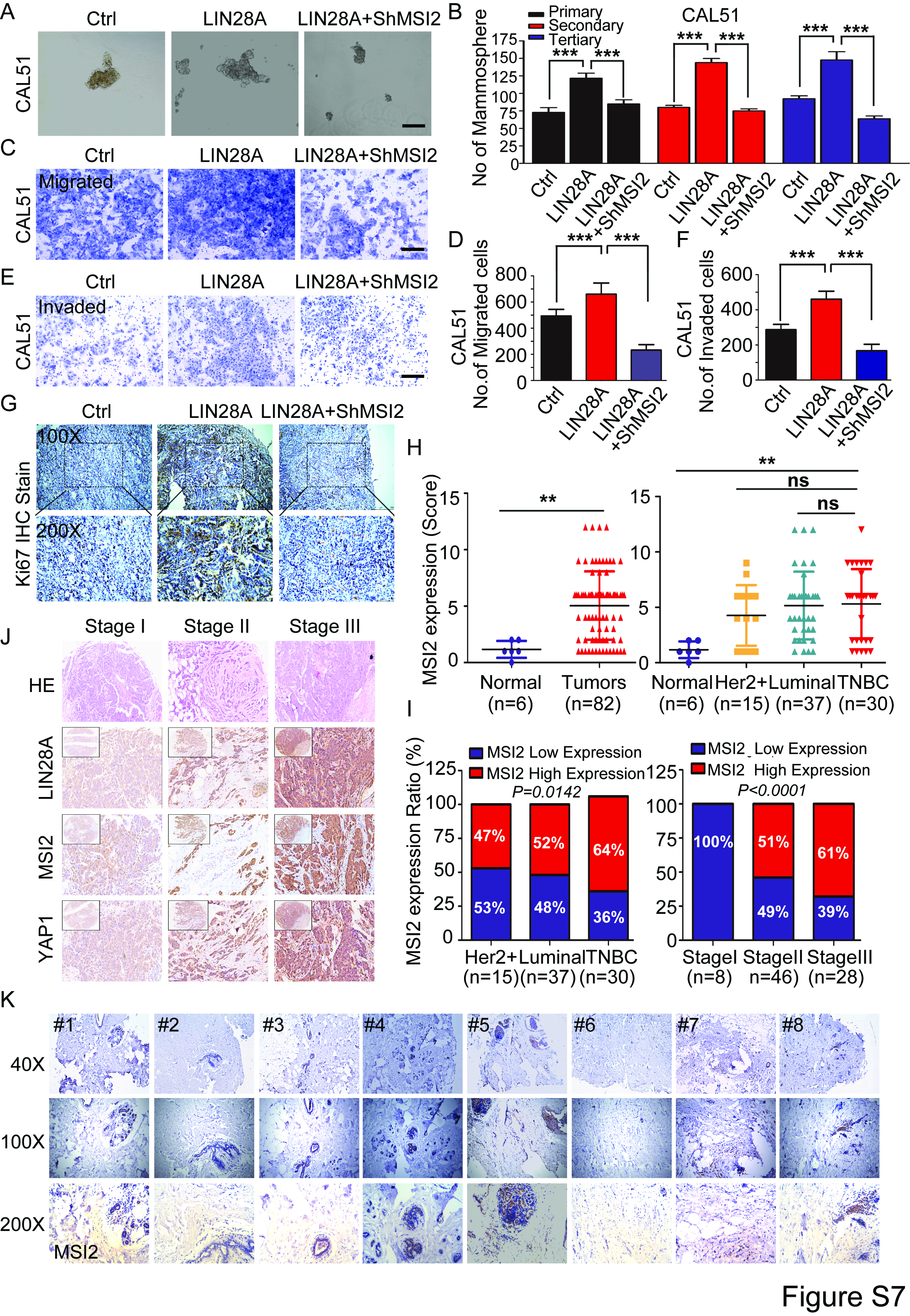

Supplement: Supplementary file 8 — Supplementary figure 7 [file 41388_2022_2198_MOESM8_ESM.tif]
