# Supplementary material for: RNA-binding protein complex LIN28/MSI2 enhances cancer stem cell-like properties by modulating Hippo-YAP1 signaling and independently of Let-7
Source: Oncogene. 2022 Jan 31;41(11):1657–72. doi: 10.1038/s41388-022-02198-w (PMC8913359; doi:10.1038/s41388-022-02198-w)
Supplement: Supplementary file 9 — Supplementary table 1 [file 41388_2022_2198_MOESM9_ESM.docx]

**Supplementary table 1: List of oligos used in this study**

| **Target gene** | **Oligo sequences** | **Experiment used** |
| --- | --- | --- |
| GAPDH | Forward: 5'-TCGGAGTCAACGGATTTGGT-3'  Reverse: 5'-TTCCCGTTCTCAGCCTTGAC-3' | QRT-PCR |
| LIN28A | Forward: 5’-CGGGCATCTGTAAGTGGTTCA-3’  Reverse: 5’-ACCCTTCCATGTGCAGCTTA-3’ | QRT-PCR |
| LIN28B | Forward: 5’-ATCTCAGAACGGTCAGGCAG-3’  Reverse: 5’-TGAAGGCCCCTTTTTGCTTTG-3’ | QRT-PCR |
| YAP1 | Forward: 5’-ACCTTGAAGCCATTCCTGGG-3’  Reverse: 5’-TCCTGCAGACTTGGCATCAG-3’ | QRT-PCR |
| TAZ | Forward: 5’-AATTCCTGCGTTTCAAGTGGG-3’  Reverse: 5’-GAAGTAGGGCGGACTGTTAGG-3’ | QRT-PCR |
| CTGF | Forward: 5’-AGGTGTGGCTTTAGGAGCAG-3’  Reverse: 5’-TCTTGATGGCTGGAGAATGC -3’ | QRT-PCR |
| CYR61 | Forward: 5’-TGGAACTGGTATCTCCACACG-3’  Reverse: 5’-TACACTGGCTGTCCACAAGG -3’ | QRT-PCR |
| TAGLN | Forward: 5’-GGCTGGTGGAGTGGATCATA-3’  Reverse: 5’-TTGGAGCCATCAGGGTACAG-3’ | QRT-PCR |
| STK3 | Forward: 5’-GCCAGAACTTTGGTCCGATGA-3’  Reverse: 5’-TCGTTGCTGTTCCTCATGTC-3’ | QRT-PCR |
| STK4 | Forward: 5’-GCACCCATTTGTCAGGAGTG-3’  Reverse: 5’-GTGCCCATCTCATCACCCAC-3’ | QRT-PCR |
| SAV1 | Forward: 5’-AAAACGAAGTGTCCAAGCCG-3’  Reverse: 5’-GGCATAAGATTCCGAAGCAGAG-3’ | QRT-PCR |
| LATS1 | Forward: 5’-GCCAGCATGAAACCAGGGA-3’  Reverse: 5’-GCCATGCCTCTGAGGAACTAA-3’ | QRT-PCR |
| LATS2 | Forward: 5’-TGTAAGGTGGACACTCACGC-3’  Reverse: 5’-CACCCACTCATTGTCTGCCT-3’ | QRT-PCR |
| MOB1 | Forward: 5'-AGCAACTCTAGGAAGTGGGA -3'  Reverse: 5'-CCACAGTGTTCACAGCAATCC-3' | QRT-PCR |
| hLIN28A-ShRNA-1 | 5’-CCGGGCACAGAATTGAGCCACAATGCTCGAGCATTGTGGCTCAATTCTGTGCTTTTTT-3’ | LIN28A knockdown |
| hLIN28A-ShRNA-2 | 5’-CCGGTAGTTGGCACTGCCATGTATCCTCGAGGATACATGGCAGTGCCAACTATTTTTT-3’ | LIN28A knockdown |
| hLIN28A-ShRNA-3 | 5’-CCGGCCTGGTGGAGTATTCTGTATTCTCGAGAATACAGAATACTCCACCAGGTTTTTT-3’ | LIN28A knockdown |
| hYAP1-ShRNA-1 | 5’-CCGGGCCACCAAGCTAGATAAAGAACTCGAGTTCTTTATCTAGCTTGGTGGCTTTTT-3’ | YAP1 knockdown |
| hYAP1-ShRNA-2 | 5’-CCGGGACCAATAGCTCAGATCCTTTCTCGAGAAAGGATCTGAGCTATTGGTCTTTTT-3’ | YAP1 knockdown |
| hYAP1-ShRNA-3 | 5’-CCGGCAGGTGATACTATCAACCAAACTCGAGTTTGGTTGATAGTATCACCTGTTTTT-3’ | YAP1 knockdown |
| hMSI2-ShRNA-1 | 5’-CCGGCCCAACTTCGTGGCGACCTATCTCGAGATAGGTCGCCACGAAGTTGGGTTTTTG-3’ | MSI2 knockdown |
| hMSI2-ShRNA-2 | 5’-CCGGCCAGCAAGTGTAGATAAAGTACTCGAGTACTTTATCTACACTTGCTGGTTTTTG-3’ | MSI2 knockdown |
