# Supplementary material for: RNA-binding protein complex LIN28/MSI2 enhances cancer stem cell-like properties by modulating Hippo-YAP1 signaling and independently of Let-7
Source: Oncogene. 2022 Jan 31;41(11):1657–72. doi: 10.1038/s41388-022-02198-w (PMC8913359; doi:10.1038/s41388-022-02198-w)
Supplement: Supplementary file 10 — Supplementary table 2 [file 41388_2022_2198_MOESM10_ESM.docx]

**Supplementary table 2: List of antibodies used in this study**

| **Target protein** | **Description** | **Company**  **Cat no.** | **Working concentration** | **Experiment used** |
| --- | --- | --- | --- | --- |
| LIN28A | LIN28 Rabbit pAb | ABclonal A6034 | 1:2000 | WB |
| LIN28B | LIN28B Rabbit pAb | ABclonal A9524 | 1:1000 | WB |
| YAP1 | [KO Validated] YAP1 | ABclonal A1002 | 1:3000 | WB |
| pS127YAP1 | Phospho-YAP1-S127 Rabbit pAb | CST #4911 | 1:1000 | WB |
| YAP/TAZ | YAP/TAZ (D24E4) Rabbit mAb | CST #8418 | 1:2000 | WB |
| Flag | Mouse anti DDDDK-Tag mAb | ABclonal AE005 | 1:3000 | WB |
| ACTIN | ACTB Monoclonal Antibody | ABclonal AC004 | 1:5000 | WB |
| C-MYC | c-Myc Rabbit mAb | ABclonal A19032 | 1:1000 | WB |
| E-Cadherin | E-Cadherin Rabbit pAb | ABclonal A11492 | 1:1000 | WB |
| N-Cadherin | N-Cadherin Rabbit pAb | ABclonal A3045 | 1:1000 | WB |
| Vimentin | Vimentin Rabbit pAb | ABclonal A11952 | 1:2000 | WB |
| Snail | Snail Rabbit pAb | ABclonal A5243 | 1:1000 | WB |
| SOX2 | [KO Validated] SOX2 Rabbit pAb | ABclonal A0561 | 1:3000 | WB |
| MSI2 | MSI2 Rabbit mAb | ABclonal A19814 | 1:1000 | WB |
| OCT4 | Oct-3/4(C-10) a mouse monoclonal antibody | Santa cruz sc-5279 | 1:500 | WB |
| NANOG | Anti-NANOG rabbit polyclonal antibody | Sangon Biotech D155241 | 1:500 | WB |
| CTGF | CTGF Rabbit Polyclonal antibody | Proteintech 23936-1-AP | 1:1000 | WB |
| Puromycin | Anti-puromycin antibody clone 12D10 | Sigma MABE343 | 1:1000 | WB |
| LIN28A | LIN28 Rabbit pAb | ABclonal A6034 | 1:1000 | IHC |
| YAP1 | [KO Validated] YAP1 | ABclonal A1002 | 1:1000 | IHC |
| MSI2 | MSI2 Rabbit mAb | ABclonal A19814 | 1:5000 | IHC |
| MYC-tag | Rabbit anti Myc-Tag pAb-C-terminal | ABclonal AE009 | 1:500 | IP |
| Rabbit IgG | HRP Goat Anti-Rabbit IgG（H+L） | ABclonal AS014 | 1:5000 | WB |
| Mouse IgG | HRP Goat Anti-Mouse IgG（H+L） | ABclonal AS003 | 1:5000 | WB |
